# In vivo characterization of glutamine metabolism identifies therapeutic targets in clear cell renal cell carcinoma

**DOI:** 10.1126/sciadv.abp8293

**Published:** 2022-12-16

**Authors:** Akash K. Kaushik, Amy Tarangelo, Lindsey K. Boroughs, Mukundan Ragavan, Yuanyuan Zhang, Cheng-Yang Wu, Xiangyi Li, Kristen Ahumada, Jui-Chung Chiang, Vanina T. Tcheuyap, Faeze Saatchi, Quyen N. Do, Cissy Yong, Tracy Rosales, Christina Stevens, Aparna D. Rao, Brandon Faubert, Panayotis Pachnis, Lauren G. Zacharias, Hieu Vu, Feng Cai, Thomas P. Mathews, Giannicola Genovese, Barbara S. Slusher, Payal Kapur, Xiankai Sun, Matthew Merritt, James Brugarolas, Ralph J. DeBerardinis

**Affiliations:** ^1^ Children’s Research Institute, University of Texas Southwestern Medical Center, Dallas, TX, USA.; ^2^ Department of Structural Biology, St. Jude Children’s Research Hospital, Memphis, TN, USA.; ^3^ Department of Radiology, University of Texas Southwestern Medical Center, Dallas, TX, USA.; ^4^ Kidney Cancer Program, Harold C. Simmons Comprehensive Cancer Center, University of Texas Southwestern Medical Center, Dallas, TX, USA.; ^5^ Department of Surgery, University of Cambridge, Cambridge, UK.; ^6^ Peter MacCallum Cancer Centre, Melbourne, Victoria 3000, Australia.; ^7^ Sir Peter MacCallum Department of Oncology, The University of Melbourne, Melbourne, Victoria 3010, Australia.; ^8^ Department of Medicine, The University of Chicago, Chicago, IL, USA.; ^9^ Department of Genitourinary Medical Oncology, MD Anderson Cancer Center, Houston, TX, USA.; ^10^ Department of Neurology and Johns Hopkins Drug Discovery, Johns Hopkins School of Medicine, Baltimore, MD, USA.; ^11^ Advanced Imaging Research Center, University of Texas Southwestern Medical Center, Dallas, TX USA.; ^12^ Department of Biochemistry and Molecular Biology, University of Florida, Gainesville, FL, USA.; ^13^ Howard Hughes Medical Institute, Chevy Chase, MD, USA.

## Abstract

Targeting metabolic vulnerabilities has been proposed as a therapeutic strategy in renal cell carcinoma (RCC). Here, we analyzed the metabolism of patient-derived xenografts (tumorgrafts) from diverse subtypes of RCC. Tumorgrafts from *VHL*-mutant clear cell RCC (ccRCC) retained metabolic features of human ccRCC and engaged in oxidative and reductive glutamine metabolism. Genetic silencing of isocitrate dehydrogenase-1 or isocitrate dehydrogenase-2 impaired reductive labeling of tricarboxylic acid (TCA) cycle intermediates in vivo and suppressed growth of tumors generated from tumorgraft-derived cells. Glutaminase inhibition reduced the contribution of glutamine to the TCA cycle and resulted in modest suppression of tumorgraft growth. Infusions with [amide-^15^N]glutamine revealed persistent amidotransferase activity during glutaminase inhibition, and blocking these activities with the amidotransferase inhibitor JHU-083 also reduced tumor growth in both immunocompromised and immunocompetent mice. We conclude that ccRCC tumorgrafts catabolize glutamine via multiple pathways, perhaps explaining why it has been challenging to achieve therapeutic responses in patients by inhibiting glutaminase.

## Teaser

Glutamine fuels the TCA cycle and amidotransferase pathways in clear cell renal cell carcinoma.

## INTRODUCTION

Renal cell carcinomas (RCC) are characterized by mutations in genes that regulate intermediary metabolism, resulting in metabolic dependencies that have been proposed as therapeutic targets (*1*). Clear cell RCC (ccRCC), the most common subtype of RCC, almost universally loses the function of the von-Hippel-Lindau (*VHL*) tumor suppressor through mutation and other mechanisms (*2*, *3*). The VHL protein is a subunit of an E3 ubiquitin ligase complex that targets the oxygen-labile α subunits of hypoxia-inducible transcription factor–1 (HIF-1) and HIF-2 for degradation; therefore, *VHL* mutation results in sustained HIF transcriptional activity (*4*). Together, HIF-1 and HIF-2 regulate the expression of many genes, including genes involved in central carbon metabolism. HIF-1 activates the expression of glucose transporters, glycolytic enzymes, lactate dehydrogenase, and pyruvate dehydrogenase kinase-1 (*PDK1*) (*5*–*8*). Expression of these genes induces a phenotype of enhanced glycolysis and suppressed pyruvate oxidation in mitochondria (*9*, *10*). Suppression of pyruvate oxidation results from PDK1’s inhibitory phosphorylation of the pyruvate dehydrogenase (PDH) complex, which converts pyruvate to acetyl–coenzyme A (CoA) in the mitochondria (*6*).

Although the metabolic effects of *VHL* loss on metabolism are well-established in cultured ccRCC cell lines, it is unclear which, if any, effects result in clinically actionable metabolic vulnerabilities. An important bottleneck in cancer metabolism studies is the relative lack of information about pathway activity in vivo. By infusing patients with cancer intraoperatively with ^13^C-labeled nutrients and examining ^13^C enrichment in metabolites extracted from the tumor after surgery, we previously characterized human tumor metabolism in several types of cancer (*11*–*13*). Infusion of [U-^13^C]glucose into a small cohort of patients with ccRCC revealed robust ^13^C labeling of glycolytic intermediates but impaired labeling of tricarboxylic acid (TCA) cycle intermediates in the tumor relative to adjacent kidney (*13*). This analysis revealed a metabolic phenotype consistent with the Warburg effect, as predicted by transcriptomic and metabolomic studies in ccRCC (*3*, *14*–*17*).

It is unknown how human ccRCCs compensate for reduced pyruvate oxidation. In culture, *VHL*-mutant RCC cells use an unusual form of glutamine metabolism to supply the TCA cycle. In this pathway, termed reductive carboxylation, the reversible isoforms of isocitrate dehydrogenases (IDH1 and IDH2) catalyze the NADPH (reduced form of nicotinamide adenine dinucleotide phosphate)–dependent carboxylation of glutamine-derived α-ketoglutarate (α-KG) to produce isocitrate and citrate (*18*, *19*). Citrate is then transported to the cytosol and cleaved by citrate lyase to produce acetyl-CoA, which is used to generate fatty acids and oxaloacetate (OAA), which gives rise to other TCA cycle intermediates and aspartate (*18*, *19*). In ccRCC cells, reductive glutamine metabolism is a consequence of *VHL* loss because reexpressing functional *VHL* suppresses the pathway (*19*, *20*). Subcutaneous xenografts derived from an established ccRCC cell line demonstrated a small amount of reductive citrate formation, raising the possibility that this pathway might contribute to central carbon metabolism in some tumors (*20*).

Identifying tumors that unequivocally require glutamine as a fuel, even from disease-relevant preclinical models, would be valuable because several inhibitors of this pathway have been evaluated as therapeutic agents. Glutaminases (GLS) and amidotransferases initiate glutamine catabolism by converting glutamine to glutamate. GLS is a mitochondrial enzyme that releases glutamine’s amide group as ammonia. CB-839 is a potent and well-tolerated GLS inhibitor with efficacy in some patients with heavily pretreated RCC (*21*, *22*), although recent combination studies failed to achieve primary clinical end points (*23*). Amidotransferases convert glutamine to glutamate while transferring glutamine’s amide nitrogen to asparagine and intermediates in the synthesis of purines, pyrimidines, hexosamines, and nicotinamide adenine dinucleotide cofactors (*24*). The experimental amidotransferase inhibitor JHU-083, a prodrug of 6-diazo-5-oxo-l-norleucine (DON), has preclinical efficacy in multiple cancer models, suppressing tumor cell growth and enhancing antitumor immunity (*25*).

To identify actionable metabolic liabilities in cancer, animal models that faithfully recapitulate the metabolic features of human tumors are needed. Access to disease-relevant models is particularly important in analyzing glutamine metabolism as many cancer cell lines use glutamine as a respiratory substrate in culture (*26*), but this does not guarantee glutamine utilization in vivo. Tumors that have been analyzed with isotope tracers generally do not use glutamine as a prominent carbon source for the TCA cycle (*27*, *28*). Here, we characterized the metabolic features of a large panel of patient-derived RCC tumorgrafts that retain clinical and genomic features of human tumors (*29*, *30*) to determine whether they consume glutamine in vivo and respond to inhibitors of glutamine metabolism.

## RESULTS

### *VHL*-mutant tumorgrafts recapitulate core metabolic features of primary human ccRCC

To identify tractable metabolic features in RCC, we performed metabolomics in 28 independent RCC tumorgraft lines (table S1) passaged orthotopically in nonobese diabetic severe combined immunodeficient (NOD-SCID) mice, as described previously (*29*, *31*). These tumorgrafts encompass the histological and clinical diversity of aggressive forms of human RCC, including 19 high-grade ccRCCs of which 65% had confirmed *VHL* mutations, 2 each of *FH*-deficient RCC and papillary RCC, 1 translocation RCC, and 4 unclassified RCC. Twenty-two of these tumors were derived from the primary site, and the rest were from either distant (five) or regional lymph node (one)metastases. Overall, 23 of 28 of the models were derived from treatment-naïve tumors. Among the ccRCC tumorgrafts, five contained sarcomatoid features associated with poor outcomes in patients (*32*).

For metabolic analysis, we orthotopically passaged each tumorgraft into multiple NOD-SCID mice. When the tumor diameter reached ~10 to 15 mm, we harvested tissues from the tumor and contralateral kidney from two to four mice from each model. All tissues were harvested within 3 min of euthanizing the mouse. We performed targeted metabolomics on one to three fragments from each tumor and kidney. A principal components analysis of 118 metabolites revealed that all RCC subtypes were metabolically distinct from the contralateral kidney, but this unsupervised analysis did not distinguish RCC subtypes from each other (fig. S1A). Supervised analysis of the 50 metabolites that best distinguished tumors from kidneys revealed heterogeneity of metabolomic signatures among the ccRCC models, although most of them clustered together (fig. S1B). As reported in ccRCC samples from patients (*16*, *17*, *33*), metabolites related to glutathione and glycolysis were among the most altered metabolites in tumors relative to kidneys. A supervised analysis (variable importance in the projection) identified elevated glutathione and other glutamine-related metabolites and decreased purine- and pyrimidine-related metabolites in ccRCC tumorgrafts (fig. S1C). Similar observations have been reported in human ccRCC (*14*, *16*, *17*), suggesting that ccRCC tumorgrafts retain several metabolic features of human disease.

**Fig. 1.**
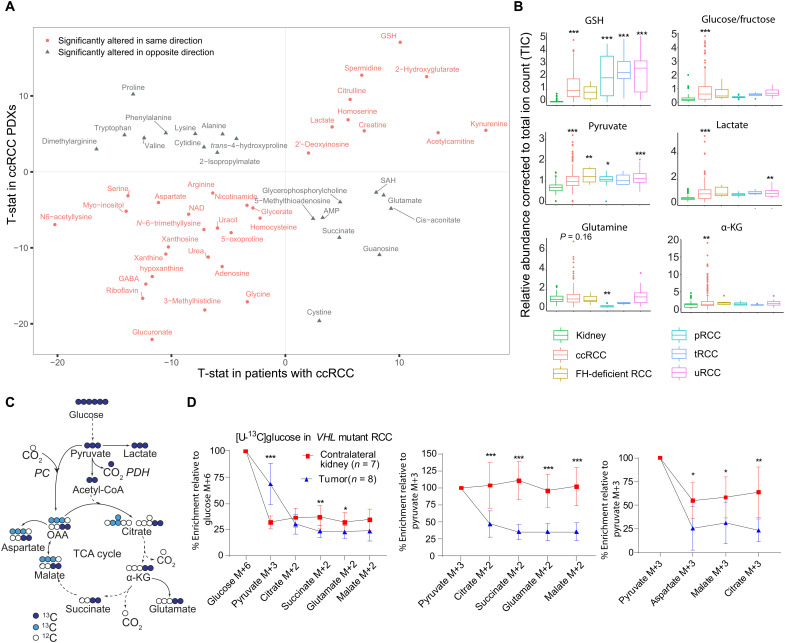
ccRCC tumorgrafts recapitulate metabolic signatures of human ccRCC. (**A**) Correlation plot of metabolites altered in both ccRCC tumorgrafts and human tumors. In both studies, differential metabolites between tumor and kidney were calculated using Student’s *t* test and false discovery rate (FDR)–corrected (*Q* < 0.05). T-statistic (T-stat) was used as a surrogate for *z* score to generate the correlation plot using ggplot in R. Positive T-stat values indicate metabolite elevation in tumors, and negative T-stat values indicate metabolite depletion in tumors. Red circles are metabolites altered in the same direction in tumorgrafts and human ccRCC, and gray triangles are metabolites altered in opposite directions. PDX, patient-derived xenograft; AMP, adenosine monophosphate; SAH, S-adenosylhomocysteine. (**B**) Box plots of relative abundance of metabolites in RCC tumorgrafts of different histological types, including ccRCC, *FH*-deficient RCC, papillary RCC (pRCC), translocation RCC (tRCC), and unclassified RCC (uRCC). One-way analysis of variance (ANOVA) coupled with pairwise *t* test in R software was used to calculate statistical significance, and *P* values were FDR-corrected. (**C**) Illustration of carbon flow from [U-^13^C]glucose. Pyruvate fuels the TCA cycle via PDH (dark blue circles) and pyruvate carboxylase (PC) (light blue circles). (**D**) Plots showing percent enrichment of metabolites relative to [U-^13^C]glucose (left), and the ratio of M+2 (middle) or M+3 (right) TCA cycle metabolites relative to pyruvate M+3 as surrogates of PDH and PC activity, respectively. Eight mice bearing three distinct orthotopic tumorgrafts (XP258, *n* = 3; XP374, *n* = 3; XP490, *n* = 2) were infused with [U-^13^C]glucose for 3 hours. M is the mass of the unlabeled metabolite. *P* values were calculated using Student’s *t* test. *P* values: *P < 0.05, **P < 0.01,***P < 0.001.

To identify robust metabolic changes shared between tumorgrafts and human cancer, we compared metabolomics data between ccRCC tumorgrafts and a large, published metabolomics dataset from human ccRCC (*17*). Unsupervised clustering of the 76 metabolites common to both datasets separated most kidney samples from tumor samples (fig. S1D). Among the 76 metabolites detected on both platforms, 51 differed between tumor and kidney in both studies, and most of these (62%) were either accumulated or depleted in both human and mouse tumors (Fig. 1A). These commonly perturbed metabolites included several related to central carbon and glutamine metabolism, including glutathione, 2-hydroxyglutarate (2-HG), lactate, and aspartate (Fig. 1A). We performed a similar analysis between the ccRCC tumorgrafts and patient tumors from UT Southwestern’s (UTSW) Kidney Cancer Program (*33*) and again observed consistent alterations in many metabolites that differentiated kidneys from tumors (fig. S2A). The data indicate that some metabolic features of human ccRCC are durable enough to withstand multiple passages in mice.

Next, we compared metabolomic alterations among the different RCC subtypes represented in the tumorgraft panel. Glutathione was markedly elevated in all subtypes relative to kidney (Fig. 1B). Glucose/fructose (our platform did not distinguish these from each other), lactate, and pyruvate from the glycolytic pathway were elevated in tumorgrafts derived from primary ccRCCs but only inconsistently in tumorgrafts from metastatic ccRCC and other RCC subtypes (Fig. 1B). Levels of several TCA cycle and related metabolites were also altered in the ccRCC tumorgrafts, with an enhanced abundance of α-KG and 2-HG and a trend toward elevated glutamine and decreased succinate (Fig. 1B and fig. S2B), as reported in human ccRCC (*17*, *33*).

### Orthotopic *VHL*-mutant tumorgrafts display reduced pyruvate oxidation relative to the nonmalignant kidney

We previously showed using isotope-labeled glucose infusions in patients that human ccRCCs display reduced contribution of pyruvate to the TCA cycle compared to patient-matched kidney tissue (*13*). To test whether the same phenotype is observed in ccRCC tumorgrafts, we infused [U-^13^C]glucose via tail vein in anesthetized NOD-SCID mice with orthotopic *VHL*-mutant ccRCC. This technique assesses the fates of carbon from plasma glucose into glucose-dependent pathways in the tissues, including glycolysis and the TCA cycle (Fig. 1C). The primary ccRCC models displayed elevated ^13^C labeling in pyruvate compared to the contralateral kidney but reduced labeling in TCA cycle–related metabolites (Fig. 1D). M+2 labeling in the TCA cycle intermediates relative to pyruvate m+3 can be used as a surrogate for carbon entry through PDH, and m+3 labeling in the TCA cycle intermediates relative to pyruvate m+3 can be used as a surrogate for carbon entry via carboxylases, e.g., by pyruvate carboxylase (Fig. 1C). The kidneys displayed good propagation of labeling from pyruvate into the TCA cycle via both pathways, but these ratios were suppressed in the tumors (Fig. 1D). This is similar to the labeling pattern observed in ccRCC patients infused with [U-^13^C]glucose during nephrectomy (*13*). Overall, in both metabolomics and isotope tracing assays, these tumorgrafts recapitulate metabolic features of human ccRCC and provide an opportunity to identify nutrients used by these tumors.

### Glutamine fuels the TCA cycle in *VHL-*mutant RCC

**Fig. 2.**
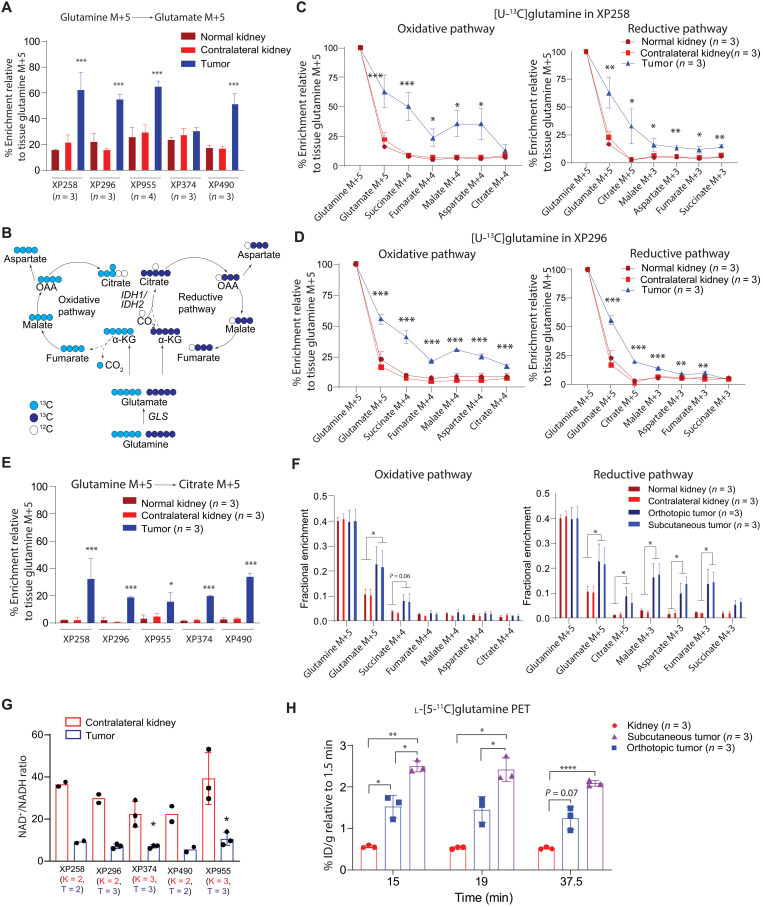
Glutamine is a carbon source for the TCA cycle in ccRCC tumorgrafts. (**A**) Relative glutamate M+5 in tumors and kidneys from mice infused with [U-^13^C]glutamine. One-way ANOVA was used to assess statistical significance. (**B**) Oxidative and reductive carbon flow from glutamine (light and dark blue circles, respectively). (**C** and **D**) TCA cycle labeling in XP258 (C) and XP296 (D) tumorgrafts, adjacent kidney (labeled as normal kidney), and contralateral kidney. The experiment was conducted in at least three mice per tumorgraft. One-way ANOVA coupled with a pairwise *t* test was used to assess significance of ^13^C enrichment between tissues. (**E**) Enrichment of citrate M+5 across all models. The experiment was conducted in a minimum of three mice per model. One-way ANOVA coupled with a pairwise *t* test was used to assess statistical significance of ^13^C enrichment in citrate M+5 between tissues. (**F**) TCA cycle intermediate labeling in orthotopic tumors, subcutaneous tumors, and kidneys. Data are from three mice implanted with both orthotopic and subcutaneous XP955 tumorgrafts. One-way ANOVA was used to assess the statistical significance of ^13^C enrichment. (**G**) NAD^+^/NADH ratios in tumorgrafts and kidneys. The number (*n*) of kidneys (K) and tumors (T) are indicated. Student’s *t* test was used to assess the significance. (**H**) ^11^C signal relative to 1.5 min (time of maximal signal in the kidney) in kidney and tumors. One-way ANOVA was used to determine statistical significance. *P* values: *P < 0.05, **P < 0.01, ***P < 0.001,**** *P *< 0.0001.

The elevation of glutamine-related metabolites and suppressed contribution of glucose to the TCA cycle suggested that glutamine is a carbon source for the TCA cycle in ccRCC tumorgrafts. To test this, we infused [U-^13^C]glutamine into mice bearing five independent *VHL*-mutant ccRCC tumorgrafts with genetic and histological heterogeneity (fig. S3A). We used an infusion method that labels 30 to 40% of glutamine and 10 to 17% of glutamate in the plasma (fig. S3B). Glutamine enrichment did not differ between the tumors and adjacent benign or contralateral kidney (fig. S3C). However, four of five tumorgrafts had elevated labeling in glutamate relative to the plasma and nonmalignant kidney, suggesting enhanced conversion of glutamine to glutamate in the tumors (Fig. 2A and fig. S3B). GLS was expressed in all tumorgrafts at levels comparable to the kidney (fig. S3D).

Next, we examined the contributions of glutamine-derived carbon into TCA cycle metabolites, assessing both the oxidative and reductive pathways of glutamine metabolism (Fig. 2B). [U-^13^C]glutamine labels glutamate and α-KG as m+5. Oxidation of α-KG results in m+4 labeling in other TCA cycle intermediates, while reductive carboxylation generates m+5 citrate and m+3 in other metabolites (*18*). Most of the tumors had elevated ^13^C labeling of α-KG compared with kidney tissues (fig. S3E). All tumors displayed elevated ^13^C labeling in at least some of the other TCA cycle intermediates as well. XP258 and XP296 demonstrated enhanced labeling along both pathways, with reductive labeling particularly evident in citrate and oxidative labeling characterizing the rest of the cycle (Fig. 2, C and D). In both of these models, oxidative labeling exceeded reductive labeling in all metabolites besides citrate, indicating flow around the TCA cycle to OAA, but suppressed formation of citrate via citrate synthase likely because of reduced PDH activity (*34*, *35*). XP955 demonstrated more prominent reductive labeling, and the other two lines had less consistent labeling around the cycle (fig. S3, F to H). All five tumors had enhanced citrate m+5 (reductive) labeling relative to the kidney, even XP374, which did not display elevated glutamate labeling relative to the kidney (Fig. 2E). The increased contribution of glutamine is notable in that glutamine catabolism in tumors analyzed by ^13^C tracing in vivo is generally low (*27*, *28*).

To confirm the reductive formation of citrate in these tumors, we infused [1-^13^C]glutamine. In this labeling scheme, ^13^C is released by α-KG dehydrogenase but retained in citrate when α-KG becomes carboxylated (fig. S4A). *VHL*-mutant tumorgrafts (XP258 and XP955) contained enhanced citrate m+1 relative to healthy kidney tissues (fig. S4A). We also used ^13^C nuclear magnetic resonance (NMR) to determine the position of ^13^C labels in malate to confirm the contribution of reductive glutamine metabolism in tumors compared to nonmalignant kidney (details in Supplementary Text and fig. S4, B to E).

Because the microenvironment influences tumor metabolism (*36*, *37*), we asked whether the site of implantation affects glutamine-dependent labeling. XP955 tumors were implanted into the subcutaneous space and renal capsules in the same mice. Then, the mice were infused with [U-^13^C]glutamine, and metabolites were extracted from tumors at both sites. Absolute ^13^C enrichment in glutamate and TCA cycle intermediates were nearly identical between subcutaneous and orthotopic tumors, with tumors at both sites demonstrating increased labeling in TCA cycle intermediates relative to nonmalignant kidney (Fig. 2F). Overall, this experiment indicates that glutamine handling in these tumors results from cancer cell–autonomous properties rather than the microenvironment.

Reductive carboxylation in cultured cells has been ascribed to an increased α-KG/citrate ratio or a shift in the cellular redox ratio to a more reduced state [i.e., decreased NAD^+^/NADH (reduced form of NAD^+^) ratio] (*20*, *35*). NAD^+^ and NADH measurements in these tumors revealed lower NAD^+^/NADH ratios than in nonmalignant kidneys (Fig. 2G and fig. S5A). Although both α-KG and citrate levels tended to be elevated in tumors, the α-KG/citrate ratio was not markedly different from the kidneys (fig. S5B).

ccRCC tumorgrafts contained elevated levels of GSH (reduced form of glutathione) and 2-HG (Fig. 1B and fig. S2B), both of which are also abundant in human ccRCC (*14*, *17*, *20*, *33*, *38*). Infusion with [U-^13^C]glutamine resulted in labeling in both metabolites (fig. S5, C and D). In GSH, the fractional enrichment was similarly low in the tumor and kidney, but the accumulation of GSH in the tumors translated into a much higher abundance of labeled GSH. Labeled 2-HG was present in the tumors but not the kidneys and was nearly all L-2HG (fig. S5, D and E), as previously reported in human ccRCC (*38*).

### l-[5-^11^C]glutamine positron emission tomography and computed tomography in ccRCC tumorgrafts

To assess glutamine metabolism using a different method, we performed positron emission tomography and computed tomography (PET/CT) with l-[5-^11^C]glutamine in mice bearing XP490 tumorgrafts in orthotopic and subcutaneous regions. Given the low soft-tissue contrast of CT, we assessed the anatomical location of the tumors and contralateral kidney using magnetic resonance imaging (MRI) (fig. S6A). The dynamic distribution of l-[5-^11^C]glutamine should reflect uptake and retention of glutamine by the tumors as long as the ^11^C label remains in the tissue. In the kidney, the time activity curve of ^11^C radioactivity [percent injected dose per gram (% ID/g)] reached its maximum (~20% ID/g) within 1.5 min of injection and then dropped to about half after 37.5 min, indicating a prominent washout of l-[5-^11^C]glutamine. This observation was similarly reported in patients with metastatic colorectal cancer (*39*). In the tumors, although the peak PET signal was lower than that in kidney, it was retained over the entire time course (fig. S6B). A significantly increased ratio of signal uptake relative to the peak time point demonstrated higher signal retention in the tumors (both orthotopic and subcutaneous) relative to kidney (Fig. 2H). This retention is consistent with glutamine’s extensive participation in metabolism in these tumors.

### IDH1 and IDH2 regulate reductive carboxylation in ccRCC

**Fig. 3.**
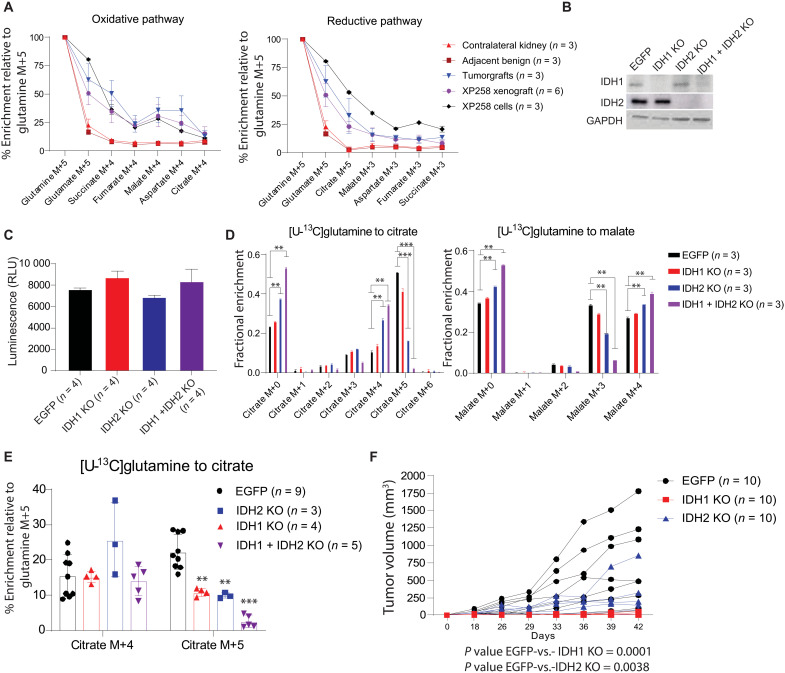
IDH1 and IDH2 regulate reductive carboxylation and ccRCC growth. (**A**) Percent enrichment of ^13^C relative to [U-^13^C]glutamine in TCA cycle from orthotopic XP258–derived orthotopic tumorgrafts, cell lines, and subcutaneous xenografts. Tumor-bearing mice were infused with [U-^13^C]glutamine for 5 hours, and XP258 cells were labeled in culture with [U-^13^C]glutamine for 4 hours. (**B**) Western blot showing the abundance of IDH1 and IDH2 in single- and double-knockout (IDH1 + IDH2 KO) cells generated using lentiviral CRISPR-Cas9. Enhanced green fluorescent protein (EGFP) is a control cell line with a guide RNA targeting EGFP. (**C**) Viable cell content in EGFP, IDH1 KO, IDH2 KO, and IDH1 + IDH2 KO XP258 cells assessed using Promega CellTiter-Glo. RLU, relative light unit. (**D**) Fractional enrichment of ^13^C-labeled isotopologues of citrate and malate in cells after 4 hours of culture with [U-^13^C]glutamine. One-way ANOVA was used to assess the significance of ^13^C enrichment in citrate and malate (*n* = 3). (**E**) Percentage enrichment of citrate M+4 and citrate M+5 relative to [U-^13^C]glutamine in EGFP, IDH1 KO, IDH2 KO, and IDH1 + IDH2 KO XP258 tumors. Mice were infused with [U-^13^C]glutamine for 5 hours. Data are from three or more xenografts generated subcutaneously in NOD-SCID mice. One-way ANOVA with a pairwise *t* test was used to determine the statistical significance. (**F**) Growth of EGFP, IDH1 KO, and IDH2 KO subcutaneous XP258 xenografts in NOD-SCID mice (*n* = 10, each cell line). P values were calculated using a mixed-effect two-way ANOVA that assessed the significance of differences in tumor growth over time between EGFP versus IDH1 KO and EGFP versus IDH2 KO tumors. *P* values: **P < 0.01, ***P< 0.001.

Reductive carboxylation is catalyzed by NADPH-dependent cytosolic IDH1 and mitochondrial IDH2 (fig. S7A) (*40*). To study the mechanism of reductive carboxylation in vivo and the role of IDH1 and IDH2 in tumorgraft growth, we generated a cell line from the *VHL*-mutant tumorgraft XP258. Like XP258 tumorgrafts, this cell line displayed both oxidative and reductive metabolism of glutamine in culture, and xenografts generated from XP258 cells after several passages in culture displayed nearly identical patterns of glutamine metabolism as the original tumorgrafts (Fig. 3A).

We next used CRISPR-Cas9 to create pools of cells lacking IDH1, IDH2, or both (Fig. 3B). XP258 cells do not form single-cell clones; therefore, we used pooled knockout (KO) cells that likely contained a small minority of cells still expressing IDH1 and IDH2. Pools lacking IDH1, IDH2, or both proliferated in the culture at the same rate as parental cells (Fig. 3C). Reductive labeling of citrate from [U-^13^C]glutamine was only marginally changed by IDH1 loss, but IDH2 loss had a more substantial effect, and reductive labeling was nearly eliminated in cells lacking both IDH1 and IDH2 (Fig. 3D). In cells lacking IDH2 or both IDH1 and IDH2, a higher fraction of citrate and malate displayed labeling through the oxidative pathway (Fig. 3D). Loss of IDH2 or both IDH1 and IDH2 also resulted in enhanced labeling of TCA cycle intermediates from [U-^13^C]glucose (fig. S7B).

We next examined the role of IDH1 and IDH2 in xenograft metabolism and growth. Xenografts from either IDH1 KO or IDH2 KO had a reduced fraction of citrate formed through reductive carboxylation, and this fraction was nearly eliminated by the loss of both enzymes (Fig. 3E). In contrast to the in vitro experiments (fig. S7B), glucose contribution to the TCA cycle remained similar across control, IDH1, and IDH2 KO tumors, with a small increase in citrate M+2 labeling in tumors lacking both IDH1 and IDH2 (fig. S7C). The loss of IDH1 or IDH2 also reduced the growth of XP258 tumors (Fig. 3F). We could not consistently generate enough palpable tumors from cells lacking both IDH1 and IDH2 to perform xenograft growth experiments despite their normal proliferation rate in culture. Examination of protein expression at the end of these experiments revealed that most tumors reexpressed at least some IDH1 and IDH2 (fig. S7D). We confirmed this in a second cell line generated from XP258 tumorgrafts, again noting xenograft growth suppression but some recovery of IDH1 and IDH2 expression by the end of the experiment (fig. S7, E to G).

### The GLS inhibitor CB-839 moderately decreases glutamine metabolism and growth in ccRCC tumorgrafts

**Fig. 4.**
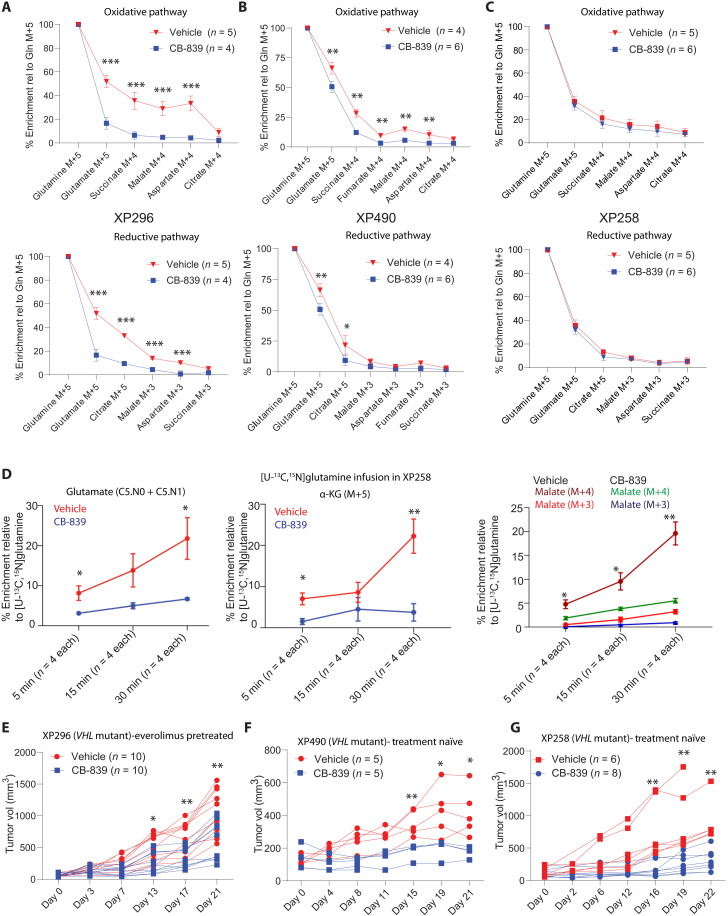
CB-839 variably decreases glutamine metabolism and ccRCC growth. (**A**) Percent enrichment of ^13^C-labeled TCA cycle intermediates relative to [U-^13^C]glutamine in the oxidative (top) and reductive (bottom) pathway in XP296 tumorgraft–bearing mice treated with CB-839 (200 mg/kg) or vehicle for 21 days. Student’s *t* test was used to assess the statistical significance of ^13^C enrichment between CB-839– and vehicle-treated tumors (*n* ≥ 4). (**B**) Similar to (A) but for XP490 tumorgrafts. (**C**) Similar to (A) but for XP258 tumorgrafts. (**D**) Percent enrichment of glutamate M+5 or M+6 {as either [U-^13^C]glutamate or [U-^13^C, ^15^N]glutamate}, M+5 α-KG and M+4 or M+3 malate in XP258 xenografts from CB-839– and vehicle-treated animals infused with [U-^13^C, ^15^N]glutamine for 5, 15, and 30 min. Student’s *t* test was used to calculate the *P* values. (**E**) Spider plot of growth of subcutaneous XP296 tumorgrafts in NOD-SCID mice treated twice daily with CB-839 (200 mg/kg) or vehicle. Treatment was started when tumors reached 100 to 200 mm^3^. Data are plotted from two independent experiments. The Student’s *t* test was used to calculate the statistical significance of tumor growth at each time point (*n* = 10, each treatment arm). (**F**) Similar to (E) but for XP490 tumorgrafts. (**G**) Similar to (E) but for XP258 tumorgrafts. *P* values: *P < 0.05, **P < 0.01, ***P < 0.001.

We next examined the effects of CB-839 on tumor metabolism and growth. We generated subcutaneous tumors from XP490, XP258, and XP296 tumorgrafts. XP490 and XP258 were generated from treatment-naïve patients, and XP296 was generated from a patient previously treated with everolimus. CB-839 decreased glutamine’s contribution to the TCA cycle along both the oxidative and reductive pathways in XP296 and XP490 but not in XP258 tumors after a 5-hour [U-^13^C]glutamine infusion (Fig. 4, A to C). CB-839 also decreased glutamine’s contribution to glutathione and L-2HG in XP296 tumors (fig. S8, A and B). To more carefully assess glutamine metabolism in XP258 tumors, we performed short (5, 15 and 30 min) infusions of [U-^13^C,^15^N]glutamine, reasoning that this would allow us to detect early differences in metabolite labeling that were no longer apparent when labeling approached the steady state. These infusions revealed low ^13^C enrichment in glutamate, α-KG, and malate in CB-839 compared to vehicle-treated tumors (Fig. 4D), indicating that CB-839 does alter glutamine metabolism in this model. All three tumorgraft models displayed modest growth suppression upon CB-839 treatment, with most tumors displaying stable volume over 3 weeks (Fig. 4, E to G). Tumor regression was not observed, and the body weights of animals were largely unaffected by CB-839 (fig. S8C).

### Amidotransferase inhibition by JHU-083 decreases glutamine metabolism and tumor growth in *VHL-*mutant ccRCC

Despite the effect of CB-839 on carbon transfer from glutamine into TCA cycle intermediates, residual glutamate labeling was noted in all tumors (Fig. 4, A to C). This was not unexpected as amidotransferases and other enzymes convert glutamine to glutamate and might compensate for GLS blockade (fig. S8D) (*24*, *41*). To examine the contribution of these reactions, we infused glutamine labeled on the amide nitrogen, [amide-^15^N]glutamine, into mice with XP296, XP258, and XP490 tumorgrafts and examined isotope enrichment in metabolites in the presence and absence of CB-839. We first assessed labeling in orotate, an intermediate in pyrimidine metabolism that acquires nitrogen from the amide position of glutamine through the activity of the trifunctional CAD (carbamoyl-phosphate synthetase-2/aspartate transcarbamylase/dihydroorotase) enzyme. Orotate was highly labeled in all tumors studied and enrichment tended to be higher in the tumors than the blood, consistent with local production (fig. S8, E to G). Orotate was also labeled in tumors treated with CB-839 (fig. S8, E to G), indicating persistent amidotransferase activity during GLS inhibition.

Other metabolites were also labeled from [amide-^15^N]glutamine. Citrulline, a urea cycle intermediate that acquires nitrogen from ammonia via the carbamoylphosphate synthetase-1 and ornithine transcarbamylase activities in liver mitochondria, was also highly labeled. However, citrulline labeling was equivalent between tumor and plasma and was suppressed by CB-839 (fig. S8, E to G). These findings suggest that unlike orotate, tumors acquire labeled citrulline primarily from the blood. CB-839 may suppress citrulline labeling because ammonia generated through GLS activity in the liver and elsewhere supplies the urea cycle.

**Fig. 5.**
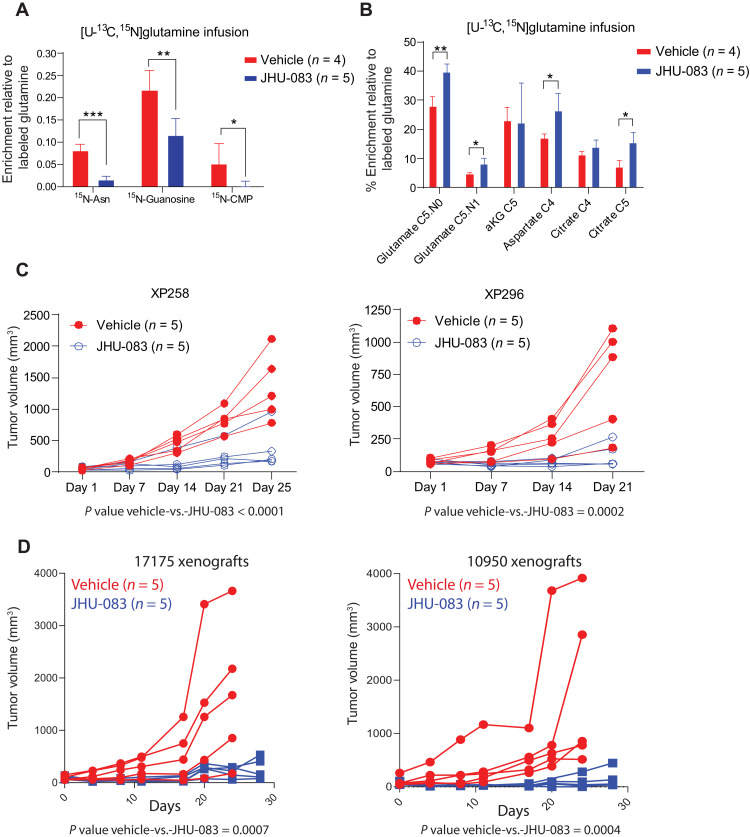
JHU-083 decreases glutamine-dependent nitrogen metabolism and ccRCC growth. (**A**) Percent enrichment of ^15^N-labeled asparagine (Asn), guanosine, and cytidine monophosphate (CMP) relative to [U-^13^C,^15^N]glutamine in XP258 tumorgrafts treated with JHU-083 (1.83 mg/kg) or vehicle for 5 days. Student’s *t* test was used to assess the statistical significance of ^15^N enrichment between JHU-083– and vehicle-treated tumors (*n* ≥ 4). (**B**) Percent enrichment of ^13^C-labeled TCA cycle intermediates relative to [U-^13^C, ^15^N]glutamine in XP258 tumorgrafts treated with JHU-083 and vehicle in (A). Student’s *t* test was used to assess the statistical significance of ^13^C enrichment between JHU-083– and vehicle-treated tumors (*n* ≥ 4). (**C**) Left: Spider plot of growth of subcutaneous XP258 tumorgrafts in NOD-SCID mice treated with JHU-083 (1.83 mg/kg) or vehicle for 25 days (5-days-on and 2-days-off treatment). Treatment was started when tumors reached ~50 mm^3^. *P* values were calculated using a mixed-effect two-way ANOVA that assessed the significance of differences in tumor growth over time between vehicle and JHU-083 in both xenografts. Right: The same as left but for XP296 tumorgrafts treated with JHU-083 (1.83 mg/kg) and vehicle for 21 days. (**D**) Growth curves of tumors in C57BL/6 mice generated from 17175 (right) and 10950 (left) cell lines treated with JHU-083 and vehicle. Mice were treated with JHU-083 (0.915 mg/kg) or vehicle for 21 days or more. *P* values were calculated using a mixed-effect two-way ANOVA that assessed the significance of differences in tumor growth over time between vehicle and JHU-083 in both xenografts. *P* values: *P < 0.05, **P < 0.01, ***P < 0.001.

We next studied the effect of amidotransferase inhibition on glutamine metabolism and growth in ccRCC xenografts. Mice with XP258 and XP296 tumors were treated with JHU-083. Infusions with [U-^13^C, ^15^N]glutamine were performed in other mice earlier in treatment to determine the metabolic effects of JHU-083. Several metabolites from pathways involving amidotransferases, including asparagine, guanosine, and cytosine monophosphate displayed suppressed labeling upon JHU-083 treatment (Fig. 5A). However, ^13^C labeling of TCA cycle intermediates was either unaffected or increased by JHU-083 (Fig. 5B). Labeling of these metabolites was suppressed by CB-839, suggesting that JHU-083’s effect on amidotransferases is more prominent than its effect on GLS under these conditions. Despite the persistent labeling of TCA cycle intermediates, JHU-083 suppressed the growth of XP258 and XP296 tumors (Fig. 5C) with only a small decrease in body weight (fig. S9A). Because JHU-083 has outstanding antitumor efficacy in some immunocompetent models (*25*), we tested it in two syngeneic RCC cell lines, 17175 and 10950, which primarily showed oxidative glutamine metabolism in vivo (fig. S9B). JHU-083 essentially eliminated the growth of xenografts derived from these cells in C57BL/6 mice, again with only mild weight loss (Fig. 5D and fig. S9C).

## DISCUSSION

Metabolic phenotypes in human tumors are incompletely defined, and we seldom know how well key metabolic features in human tumors are recapitulated in mice. This presents a challenge in using mouse models to identify metabolic liabilities in human tumors. Human ccRCC metabolism has been well-characterized through metabolomics and isotope-labeling studies. These studies suggest that human ccRCC tumors share metabolic features with *VHL*-deficient ccRCC cell lines, including enhanced glycolysis and suppressed pyruvate oxidation (the Warburg effect) (*13*, *14*, *16*, *17*). We extended these comparisons here by characterizing metabolism in a large series of orthotopic patient–derived tumorgrafts that retain genomic and histological features of human RCC (*30*). ccRCC-derived tumorgrafts display metabolic features of human ccRCC, including suppressed contributions of glucose to TCA cycle intermediates and accumulation of glutamine-derived metabolites, such as glutathione and L-2HG. Using two types of tracer studies, [^13^C]glutamine infusion and dynamic [^11^C]glutamine PET, we find that glutamine is a carbon source for intermediary metabolism in ccRCC tumorgrafts relative to nonmalignant kidney. The PET data indicate retention of glutamine carbon (specifically, carbon 5) within the tumors. Whether this primarily reflects glutamine’s contribution to the TCA cycle, or to glutathione, proteins, and other macromolecules, is unknown. We also note that glutamine metabolism is an intrinsic characteristic of these tumors rather than simply a consequence of the microenvironment as both ^11^C and ^13^C labeling features were similar between orthotopic and subcutaneous sites. A recent study that used radiotracers to compare glutamine uptake between cancer cells and immune cells in the tumor microenvironment also concluded that glutamine is preferentially taken up and retained by malignant cells (*42*).

Our ^13^C labeling approach reports fractional enrichment of metabolites downstream of the tracer after several hours of exposure to ^13^C-glutamine. Therefore, a limitation of the approach is that it reports glutamine’s metabolic fates but not the rates of glutamine-dependent pathways, including glutamine oxidation and reductive carboxylation. Nevertheless, glutamine’s prominent contributions to several pathways in the tumors compared to the kidneys raised the possibility of vulnerabilities associated with these pathways. We confirmed this by blocking GLS and amidotransferases. Combining these loss-of-function experiments with isotope tracing is informative because it provides direct evidence that the target enzyme contributes to the pathway in vivo.

Numerous studies in cultured cells have demonstrated enhanced reductive labeling of TCA cycle intermediates under conditions of *VHL* loss, hypoxia, PDH suppression, or defects in the TCA cycle or electron transport chain (*18*, *19*, *34*, *35*). A small amount of reductive carboxylation was also observed in xenografts of *VHL*-deficient UMCR3 cells, and growth of these tumors was suppressed by BPTES, a preclinical GLS inhibitor (*20*). The tumors in the current study generally show combined oxidative and reductive labeling, with the latter resulting in citrate m+5 during infusion with [U-^13^C]glutamine. This phenomenon may be related to PDH suppression as a reduced citrate:α-KG or NAD^+^/NADH ratio increases the appearance of reductively labeled citrate (*20*, *35*). We do not know whether these tumorgrafts contain a net reductive flux from α-KG to citrate, but the presence of citrate m+5 prompted us to examine the role of IDH1 and IDH2. Both IDH1 and IDH2 contribute to citrate m+5 labeling in vivo. Although these enzymes are dispensable for cell growth in culture, the loss of either one reduces patient-derived xenograft (PDX) growth. At present, we do not know whether reductive citrate formation per se is the key IDH1/2 function in vivo or whether these enzymes are required for survival during redox stress or glucose deprivation in vivo, as reported in other models of IDH1/2 dependence (*43*, *44*). However, the data suggest that dual targeting of these enzymes could produce a therapeutic effect in a subset of ccRCC tumors that demonstrate reductive carboxylation in vivo. A compound that inhibits the oncogenic, mutant forms of both IDH1 and IDH2 has been developed (*45*), so perhaps potent inhibition of both wild-type enzymes could be achieved with a single compound. We also note that although IDH1/2 KO suppresses the growth of *VHL*-mutant XP258 tumorgrafts, it is unclear at present whether *VHL* loss is sufficient to induce IDH1/2 dependence in vivo.

Because glutamine is a prominent carbon and nitrogen source, there has been a sustained interest in suppressing glutamine catabolism in tumors. Several recent studies have shown preclinical efficacy of targeting glutamine metabolism in vivo by inhibitors of glutamine transporters, GLS, and amidotransferases (*25*, *41*, *46*–*50*). Phase 1 dose-escalation clinical studies with CB-839 demonstrated good tolerability of the drug and stable disease or partial response in some patients with RCC (*22*). However, the CANTATA (NCT03428217) phase 3 trial revealed that combining CB-839 with cabozantinib in patients with advanced, metastatic RCC pretreated with checkpoint inhibitors or antiangiogenic therapies did not improve outcomes over cabozantinib alone (*51*). Our data indicate that tumor GLS is inhibited by CB-839 and that the response to GLS inhibition may be dependent on how other pathways compensate for GLS loss. GLS expression was similar between kidney and tumors, implying that factors beyond protein abundance (e.g. substrate availability, posttranslational modifications, etc.) account for glutamine’s higher contribution to the TCA cycle in the tumors. This also emphasizes that direct analysis of tumor metabolism by isotope tracing can uncover metabolic liabilities that would not be obvious from gene/protein expression studies. In this case, isotope tracing also provided some insight into how tumors resist CB-839. Upon treatment with CB-839, ccRCC tumorgrafts derived from treatment-naïve primary tumors display modest growth suppression and metabolic derangements. All tumors contained ^13^C-labeled glutamate and downstream metabolites despite CB-839 therapy, indicating persistent glutamine metabolism in the tumors. Transfer of ^15^N from [amide-^15^N]glutamine or [U-^13^C,^15^N]glutamine to products of amidotransferase reactions, including asparagine and intermediates from nucleotide metabolism, indicates that this family of enzymes enables residual glutamine catabolism in CB-839–treated tumors.

Since the early 1950s, clinical studies in several kinds of cancer reported stabilization or remission in some patients treated with low-dose DON (*52*, *53*). However, clinical development was halted because of inconsistent response and gastrointestinal toxicity in dose-escalation studies (*54*). The recent development of JHU-083, a chemically modified prodrug of DON with selective activation in the tumor microenvironment and a reduced toxicity index, has suggested an alternative approach to broad inhibition of glutamine catabolism in cancer. In syngeneic tumors, JHU-083 impairs glutamine catabolism in cancer cells and produces a nutrient microenvironment more amenable to the antitumor effects of cytotoxic T cells and other immune cells (*25*, *55*, *56*). JHU-083’s efficacy in the immunodeficient tumorgraft models described here may underestimate its overall efficacy in tumors with robust glutamine catabolism. This is particularly relevant for ccRCC, a highly immunogenic tumor type with clinical responses to immunotherapy (*57*, *58*). Our isotope-tracing experiments with [U-^13^C,^15^N]glutamine showed decreased ^15^N enrichment in asparagine and nucleotide intermediates upon JHU-083 treatment. Although several studies have reported that JHU-083 decreases GLS activity in tumors (*55*, *59*), the effect appears to be different in ccRCC tumorgrafts. In our models, ^13^C labeling of TCA cycle intermediates is GLS dependent and inhibited by CB-839. As expected, this drug does not impair amidotransferase reactions. On the other hand, JHU-083 inhibits amidotransferase reactions but not ^13^C labeling of TCA cycle intermediates in steady-state isotope infusion experiments, suggesting that its effect on GLS in these models is modest compared to CB-839. However, both CB-839 and JHU-083 impair tumor growth. To maximize the therapeutic benefit and durability in ccRCC, it may be necessary to impair amidotransferases and GLS concurrently. Developing a strategy with an acceptable therapeutic index would likely benefit from defining which amidotransferase activities are most important for ccRCC growth.

## MATERIALS AND METHODS

### PDXs (tumorgrafts)

All mouse experiments were approved by the UTSW Institutional Animal Care and Use Committee (IACUC). All tumorgrafts used in this study were preestablished PDXs. The process of obtaining patient tissue and transplanting it into mice is part of protocols approved by the UTSW Institutional Review Board and IACUC. For tumor passaging and metabolomics analysis, tumorgrafts were passaged in the left kidney of 4- to 8-week-old male/female NOD-SCID mice, as described previously (*29*–*31*). Once the tumor was palpable and close to ~10 mm in diameter, mice were euthanized to collect tumors in ice-cold Hanks’ balanced salt solution buffer with 1% penicillin-streptomycin. For tumor passaging, tumor tissues were dissected aseptically into ~8 mm^3^ of tissues. Tumor pieces were freshly implanted into the left kidney of male or female mice, and the mice were monitored periodically for tumor growth by palpating the implanted flank. For subcutaneous tumor studies, resected orthotopic tumors were dissected into small pieces (~64 mm^3^) and surgically implanted subcutaneously in the right hind region of the mice. Experiments with both orthotopic and subcutaneous tumors were conducted by surgically implanting tumors simultaneously in the left kidney and right hind region of the mice. For the characterization of glutamine metabolism, we focused on PDXs that grew to sufficient tumor size (within 60 days or less) for metabolic and therapeutic experiments. The PDXs can be provided by J.B. pending scientific review and a completed material transfer agreement. Requests for the PDXs should be submitted to J.B.

### Metabolomics

For tissue metabolomics, mice were euthanized and tissues were collected within 3 min in liquid nitrogen to minimize metabolite degradation. Tumor and kidney tissues from mice were dissected on dry ice, and ~50 mg of the tissues was placed in 1 ml of ice-cold 80% MeOH solution in high-performance liquid chromatography (HPLC)–grade water. Tissues were homogenized using a handheld automatic sonicator and subjected to three freeze-thaw cycles between liquid nitrogen and a 37°C water bath. Supernatants were collected by centrifuging the homogenized solution at 10,000 rpm for 10 min to remove tissue debris, and then, the supernatants were evaporated overnight in a SpeedVac concentrator (Thermo Savant). Dried samples were reconstituted in 100 μl of 0.03% formic acid in HPLC-grade water, vortexed, and centrifuged, and the supernatant was transferred in LC–mass spectrometry (LC-MS) glass vials containing sample inserts. As described previously (*60*), LC-MS was performed using an AB QTRAP 5500 liquid chromatography/triple quadrupole mass spectrometer (Applied Biosystems SCIEX, Foster City, CA). Chromatograms and peak area of each metabolite were reviewed and integrated using MultiQuant software version 2.1 (Applied Biosystems SCIEX, Foster City, CA). Each metabolite’s peak area was selected from either positive or negative modes depending on previously run standards. Peak areas were corrected to blank samples and then normalized to the total ion count (TIC) of that sample to correct for variation introduced by sample handling and instrumentation. TIC-normalized data were log-transformed and median-normalized for multivariate analyses using Metaboanalyst 4.0 (*61*) (www.metaboanalyst.ca). Additional statistical analyses were performed using one-way analysis of variance (ANOVA) and Student’s *t* test in R software.

### Isotope infusions

Isotope infusion experiments were conducted when tumor size was between 10 and 50 mm in diameter for orthotopic tumors and 0.5 to 1 cm in diameter for subcutaneous tumors. When the tumors reached these sizes, the mice were fasted for 16 hours and infused in the morning with glucose for 3 hours or glutamine for 5 hours. The mice were anesthetized on a heating pad, and a catheter (connected to the infusion solution and pump) was inserted in the lateral tail vein. For [U-^13^C]glucose infusions, we prepared a 750 μl of saline solution containing 2.48 g of isotope-labeled glucose per kilogram of body weight. An initial bolus of 125 μl/min was delivered under anesthesia for 1 min, followed by 2.5 μl/min of continuous infusion for 3 hours. For glutamine infusions, we prepared 1500 μl of a saline solution containing heparin (50 U/ml) with 1.725 g of isotope-labeled glutamine per kilogram of body weight. An initial bolus of 150 μl/min was delivered under anesthesia for 1 min, followed by 2.5 μl/min of continuous infusion for 5 hours. Mice were kept under anesthesia throughout the experiment, and blood samples were collected at regular intervals via retro-orbital puncture using microcapillary tubes. At the end of the infusion, mice were euthanized, and tumors and other organs were harvested and snap-frozen in liquid nitrogen. End point blood samples were collected from the heart with an insulin syringe.

For the time-course study with [U-^13^C,^15^N]glutamine, we first generated xenografts from XP258 cell lines implanted bilaterally in NOD-SCID mice. Once the tumors were of a measurable size, we randomized the animals for seven doses of CB-839 (200 mg/kg twice daily) or vehicle treatment. Isotope infusions were performed on the last day of treatment using a protocol modified from a reported method (*62*). Specifically, the priming dose of glutamine [100 + 1.6*(weight in g) μl]/min was delivered under anesthesia through a tail vein catheter. Infusion rates were then adjusted to 0.1 μl x(weight in g)/min. Mice were euthanized, and tissues were collected for MS analysis at 5, 15, and 30 min after the infusion.

### Gas chromatography–mass spectrometry

Blood samples from all infusion experiments were kept on ice until the last sample was collected. Plasma was collected from blood samples through centrifugation at 5000 rpm for 5 min at 4°C. While 10 μl of plasma was directly resuspended in ice-cold 80% MeOH in water, tissues samples of about 5 to 20 mg were homogenized in 80% MeOH in water and subjected to three cycles of free-thaw in liquid nitrogen and a 37°C water bath. Tissue and plasma extracts were centrifuged at 10,000*g* for 10 min at 4°C to separate macromolecules and cell debris. The supernatants were vacuum-dried overnight. The dried samples were reconstituted in 40 μl of pyridine containing methoxyamine (10 mg/ml). The samples were vortexed, transferred to gas chromatography–MS (GC-MS) glass vials with inserts, and heated for 10 min at 70°C. Next, we added 80 μl of the derivatization agent [*N*-(*tert*-butyldimethylsilyl)-*N*-methyltrifluoroacetamide] and heated the samples for 1 hour at 70°C on a heating block. Samples were then loaded into an autoinjector, and 1 μl of each sample was injected for analysis. Samples were run on an Agilent 6890 gas chromatograph coupled to either an Agilent 5973 N Mass Selective Detector or an Agilent 5975C Inert XL Mass Selective Detector. The resulting chromatogram and mass isotopolog distributions were analyzed in Agilent MSD ChemStation Data-Analysis software. The area under the curve for mass isotopolog was corrected for natural abundance using in-house methods run in MATLAB.

### Liquid chromatography–mass spectrometry

For the quantification of NAD, NADH^+^, and α-KG and the analysis of metabolites labeled from [amide-^15^N]glutamine, or [U-^13^C,^15^N]glutamine, we used a hydrophilic interaction liquid chromatography (HILIC) column–based separation of metabolites on a Vanquish UHPLC coupled to a QExactive HF-X hybrid quadrupole orbitrap high-resolution mass spectrometer from Thermo Fisher Scientific (Bremen, Germany), as described previously (*63*). Briefly, ~10 to 20 mg of tissue samples were extracted in 80% acetonitrile, homogenized, subjected to three freeze-thaw cycles as described above, and cleared of macromolecules by centrifugation. Protein concentration was determined in each supernatant using the bicinchoninic acid (BCA) assay, and equivalent aliquots of protein-normalized samples were injected into the instrument. The chromatogram was analyzed as described previously (*52*) using TraceFinder software from Thermo Fisher Scientific. The ^13^C- and ^15^N-labeled data were corrected for natural abundance using IsoCorrectoR (*64*).

For targeted GSH quantification, metabolites were extracted from tissues homogenized in 80% acetonitrile solution containing *N*-ethylmaleimide (0.3 M), followed with three freeze-thaws. The samples were centrifuged, and the supernatant was analyzed with a SCIEX QTRAP 5500 LC/triple quadrupole mass spectrometer. We performed separation of metabolites on a SeQuant zic-pHILIC polymeric HPLC column (150 mm by 2 mm) in a Nexera Ultra-High-Performance Liquid Chromatography system (Shimadzu Corporation). Mobile phase solvents were 10 mM ammonium acetate aqueous [pH 9.8 adjusted with ammonia water (A) and pure acetonitrile (B)]. The gradient elution was as follows: 0 to 18 min, linear gradient 90 to 55% B and 18 to 20 min, linear gradient 55 to 30% B, 20 to 25 min, 30% B, 25 to 27 min, linear gradient 30 to 90% B; then, the column was reconditioned for 6 min using 90% B. The flow rate was 0.2 ml/min, and the column was operated at 40°C. Multiple reaction monitoring (MRM) was used to check *N*-ethylmaleimide–derivatized GSH, 433/304 (M+0, CE: 19 V), 438/304 (M+5, collision energy: 19 V).

### Measurement of D-2HG and L-2HG in tissues

Metabolites were extracted from the tissues samples with 80% methanol-water solution, and the resulting supernatant was dried in a SpeedVac. Dried pellet from unlabeled samples was mixed with [U^13^C]-D/L-2HG (internal standard for unlabeled samples, Cambridge Isotope Laboratories, 10 ng in 10 μl of acetonitrile). The mixture (or pellet from labeled samples) was dissolved in a diacetyl-l-tartaric anhydride (DATAN) solution (90 μl, 50 mg/ml in freshly mixed 80% acetonitrile/20% acetic acid, DATAN, Acros Organics). The solution was sonicated, warmed to 75°C for 30 min, cooled to room temperature, and then centrifuged again to collect the supernatant. The supernatant was dried in a SpeedVac, and the pellet was reconstituted into 1.5 mM ammonium formate aqueous solution with 10% acetonitrile (100 μl). For [U-^13^C]glutamine tracing samples, derivatized [U^13^C]-D/L-2HG was used as a standard to identify the derivatized peaks. LC-MS analysis was performed on an AB Sciex 5500 QTRAP LC/mass spectrometer (Applied Biosystems SCIEX) equipped with a triple quadrupole/iontrap mass spectrometer with electrospray ionization interface and controlled by AB Sciex Analyst 1.6.1 software. Waters Acquity UPLC HSS T3 column (150 × 2.1 mM, 1.8 μM) was used for separation. Solvents for the mobile phase were 1.5 mM ammonium formate aqueous [pH 3.6 adjusted with formic acid (A), and pure acetonitrile (B)]. The gradient elution was as follows: 0 to 12 min, linear gradient 1 to 8% B and 12 to 15 min, 99% B; then, the column was washed with 99% B for 5 min before reconditioning it for 3 min using 1% B. The flow rate was 0.25 ml/min, and the column was operated at 35°C. MRM was used to check 2-HG–diacetyl tartrate derivatives: 363/147 (M+0, CE: −14 V); 368/152 (M+5, CE: −14 V). This method was modified and adapted from previously published work (*65*).

### ^13^C NMR spectroscopy

Kidney (43 to 107 mg) and tumor (220 to 462 mg) samples were subjected to extraction in ice-cold acetonitrile-isopropanol-water by adding the solvent to preweighed tissue samples. Zirconium beads were added to the sample suspension, and the samples were homogenized using a Fastprep-24 (MP Biomedicals, California, USA). Homogenization was carried out in a “20 seconds ON – 5 minutes OFF” cycle to avoid overheating extracts and repeated at least four times. The homogenates were centrifuged at 10,000*g* for 30 min at 4°C, and the supernatant was lyophilized at room temperature in a SpeedVac system (Thermo Fisher Scientific, Waltham, MA). The dried sample was dissolved in a 1:1 acetonitrile/water mixture, centrifuged at 10,000*g* for 30 min at 4°C, and lyophilized again.

Samples were prepared for NMR analysis by dissolving lyophilized powder in 54 μl of 50 mM sodium phosphate buffer in D_2_O containing 2 mM EDTA. An internal standard (6 μl) containing 0.02% (w/v) NaN_3_ and 0.5 mM deuterated sodium 3–trimethylsilyl–1–propanesulfonate was added to the sample solution. The solution was vortexed thoroughly and centrifuged at 10,000*g* for 5 min, and 54 μl of the supernatant was loaded into a 1.5-mm NMR tube. All NMR spectra were acquired using a 14.1-T NMR magnet equipped with a home-built high-temperature superconducting) probe (*66*). Parameters included an acquisition time of 1.5 s, a spectral width of 240 parts per million (ppm), and a ^1^H decoupling field strength of 4800 Hz. The spectra were processed (zero-filled to 131,072 points, 0.8-Hz exponential line broadening, and polynomial or spline baseline correction as necessary) and referenced by setting the singlet resonance of taurine (N-C1) to 48.4 ppm. Peaks of interest were fitted to a mixed Gaussian/Lorentzian shape, and area under the peaks was obtained.

### l-[5-^11^C]glutamine PET/CT and MRI

A 9 cm-long catheter of 0.38-mm inner diameter fitted with a 27-gauge needle was inserted into the tail of a tumor-bearing mouse for intravenous injection of l-[5-^11^C]-glutamine (~ 5 MBq per mouse). Dynamic PET data acquisition was performed on a Mediso NanoScan PET/CT System (Mediso, USA) immediately after the injection for up to 40 min, followed by a CT scan with 720 projections. The images were reconstructed using the manufacturer’s software and quantitatively analyzed by Inveon Research Workplace (Siemens, USA). MRI was performed on a 1 Tesla desktop MR scanner (M2 Compact, Aspect Imaging, Shoham, Israel) using a mouse volume coil. T2-weighted imaging was performed with a fast spin-echo (repetition time/time to echo = 2500/80 ms) sequence with the mouse in a prone position.

### Cell lines derived from XP258 tumorgrafts

Orthotopic tumors were dissected and collected in ice-cold 20 ml of transport media [minimum essential medium, gentamycin (50 μg/ml), fungizone (2.5 μg/ml), and 1× penicillin-streptomycin]. The tissues were washed three times in 15 ml of transport media and minced into small pieces using sterile Swan-Motron blades. Minced tissue was resuspended in 15 ml of enzyme mix (transport media, collagenase, hyaluronidase, and deoxyribonuclease IV) and incubated for 1 to 2 hours in 5% CO_2_ in a 37°C incubator. Enzyme-digested tissue samples were filtered to separate cells from debris and larger tissue pieces. Freshly obtained cells were cultured in high-glucose Dulbecco’s modified Eagle’s medium (DMEM) supplemented with 10% fetal bovine serum (FBS), 1% penicillin-streptomycin, 1× nonessential amino acids, hydrocortisone (0.8 mg/ml), and epidermal growth factor (10 μg/ml).

### Syngeneic RCC cell lines

We used 17175 and 10950 cell lines, which were generated and provided by the laboratory of G.G., MD Anderson Cancer Center. The request to obtain these cells should be directly addressed to G.G. These cells were plated in 0.1% gelatin–coated plates (catalog no. ES-006-B, Merck Millipore) in high glucose–containing DMEM with 20% FBS and 1% penicillin-streptomycin. To generate tumors, we injected 1 million cells of each cell line mixed 50:50 (v/v) with Matrigel into the flanks of C57BL/6 mice.

### KO of IDH1 and IDH2 using CRISPR-Cas9

IDH1 and IDH2 KO pools were generated using the CRISPR-Cas9 system, as described previously (*44*). Briefly, IDH1 single guide RNA (sgRNA) (ACGTGGAATTGGATCTACAT) and IDH2 sgRNA (ATGAGATGACCCGTATTATC) were cloned into lentiCRISPR V2 system following a published protocol (*67*). These vectors were transfected into human embryonic kidney 239T cells, and medium containing viral particles was collected at 48 and 72 hours and filtered through a 0.45 μM filter. The tumorgraft-derived XP258 cell lines were transduced with media containing viral particles and then selected in puromycin (2 μg/ml) to generate pools of modified cells. IDH1/2 double-KO XP258 cells were generated by transducing IDH2 KO cells with viral particles carrying the IDH1 sgRNA.

### Immunoblotting

Cells and tissues were homogenized in radioimmunoprecipitation assay buffer containing protease and phosphatase inhibitors. Supernatants were collected by centrifugation at 14,000 rpm for 20 min at 4°C. Protein concentrations were determined using the BCA protein assay. Protein lysates were resolved in SDS–polyacrylamide gel electrophoresis and transferred to methanol-activated nitrocellulose membranes. The membrane was first blocked with 5% milk and then incubated overnight with primary antibodies against GLS (Thermo Fisher Scientific, catalog no. PA5-40135), IDH1 (Cell Signaling Technology, catalog no. 8137 or Abcam catalog no. ab239606), IDH2 (Abcam, ab55271), β-actin (catalog no. 5125S), or glyceraldehyde-3-phosphate dehydrogenase (catalog no. 8884S) from Cell Signaling Technology.

### CB-839 and JHU-083 treatment

Calithera Inc. (San Francisco, CA) provided the solution for CB-839 and vehicle for in vivo experiments. CB-839 (200 mg/kg) or vehicle in 200 μl of solution was administered orally every 12 hours for 21 to 22 days, as described previously (*48*). JHU-083 was provided by B.S.S. JHU-083 was diluted in 50 mM Hepes-buffered saline such that 100 μl contained JHU-083 (1.83 mg/kg). Mice with ccRCC tumorgrafts were administered either 100 μl of JHU-083 or vehicle, whereas the mice with syngeneic cell line–derived tumors were administered 50 μl of JHU-083 or vehicle. Mice were dosed with JHU-083 or vehicle via intraperitoneal injection according to a regimen of 5 days on and 2 days off. Tumor growth and body weight were monitored twice weekly. Tumor volume was measured using electronic calipers and calculated using the formula length × (width^2^)/2. Infusion of [U-^13^C]glutamine and metabolomics were conducted at the end of 21 days of the CB-839 treatment. Infusion with [amide-^15^N]glutamine, [U-^13^C,^15^N]glutamine, and [U-^13^C]glucose were conducted after five to seven doses of the drugs (JHU-083 or CB-839) or vehicle.

### Isotope tracing in vitro

One million cells were plated overnight for each experiment. For isotope-tracing experiments, cells were washed with phosphate-buffered saline and then fed with medium containing 100% isotope-labeled tracer of interest with 10% dialyzed FBS. [U-^13^C]glucose and [U-^13^C]glutamine labeling was performed over 3 to 4 hours. After the experiment, the cells were washed once in ice-cold saline solution, and 1 ml of 80% methanol solution was added. The cells were collected by scraping and subjected to three freeze-thaw cycles followed by centrifugation to remove debris. The supernatant was vacuum-dried overnight in a SpeedVac and reconstituted for GC-MS analysis as described above.

### Statistical analysis

Metaboanalyst 4.0 (*61*) and R software (www.metaboanalyst.ca) were used to conduct statistical analysis of the metabolomics data. R software and PRISM were used to conduct one-way ANOVA and Student’s *t* test, respectively. Data visualization used both PRISM and R software. Descriptions of individual statistical analyses can be found in the figure legends.

## Notes

View/request a protocol for this paper from *Bio-protocol*.

## References

[1] W. M. Linehan, L. S. Schmidt, D. R. Crooks, D. Wei, R. Srinivasan, M. Lang, C. J. Ricketts,The metabolic basis of kidney cancer. Cancer Discov. 9,1006–1021 (2019). 31088840 10.1158/2159-8290.CD-18-1354

[2] X. Ma, K. Yang, P. Lindblad, L. Egevad, K. Hemminki,VHL gene alterations in renal cell carcinoma patients: Novel hotspot or founder mutations and linkage disequilibrium. Oncogene 20,5393–5400 (2001). 11536052 10.1038/sj.onc.1204692

[3] Cancer Genome Atlas Research Network,Comprehensive molecular characterization of clear cell renal cell carcinoma. Nature 499,43–49 (2013). 23792563 10.1038/nature12222 PMC3771322

[4] D. Tarade, M. Ohh,The HIF and other quandaries in VHL disease. Oncogene 37,139–147 (2018). 28925400 10.1038/onc.2017.338

[5] G. L. Semenza, B. H. Jiang, S. W. Leung, R. Passantino, J. P. Concordet, P. Maire, A. Giallongo,Hypoxia response elements in the aldolase A, enolase 1, and lactate dehydrogenase A gene promoters contain essential binding sites for hypoxia-inducible factor 1. J. Biol. Chem. 271,32529–32537 (1996). 8955077 10.1074/jbc.271.51.32529

[6] J. W. Kim, I. Tchernyshyov, G. L. Semenza, C. V. Dang,HIF-1-mediated expression of pyruvate dehydrogenase kinase: A metabolic switch required for cellular adaptation to hypoxia. Cell Metab. 3,177–185 (2006). 16517405 10.1016/j.cmet.2006.02.002

[7] B. L. Ebert, J. D. Firth, P. J. Ratcliffe,Hypoxia and mitochondrial inhibitors regulate expression of glucose transporter-1 via distinct Cis-acting sequences. J. Biol. Chem. 270,29083–29089 (1995). 7493931 10.1074/jbc.270.49.29083

[8] G. L. Semenza, P. H. Roth, H. M. Fang, G. L. Wang,Transcriptional regulation of genes encoding glycolytic enzymes by hypoxia-inducible factor 1. J. Biol. Chem. 269,23757–23763 (1994). 8089148

[9] G. L. Semenza,HIF-1: Upstream and downstream of cancer metabolism. Curr. Opin. Genet. Dev. 20,51–56 (2010). 19942427 10.1016/j.gde.2009.10.009 PMC2822127

[10] G. L. Semenza,HIF-1 mediates the Warburg effect in clear cell renal carcinoma. J. Bioenerg. Biomembr. 39,231–234 (2007). 17551816 10.1007/s10863-007-9081-2

[11] E. A. Maher, I. Marin-Valencia, R. M. Bachoo, T. Mashimo, J. Raisanen, K. J. Hatanpaa, A. Jindal, F. M. Jeffrey, C. Choi, C. Madden, D. Mathews, J. M. Pascual, B. E. Mickey, C. R. Malloy, R. J. DeBerardinis,Metabolism of [U-13 C]glucose in human brain tumors in vivo. NMR Biomed. 25,1234–1244 (2012). 22419606 10.1002/nbm.2794 PMC3406255

[12] C. T. Hensley, B. Faubert, Q. Yuan, N. Lev-Cohain, E. Jin, J. Kim, L. Jiang, B. Ko, R. Skelton, L. Loudat, M. Wodzak, C. Klimko, E. McMillan, Y. Butt, M. Ni, D. Oliver, J. Torrealba, C. R. Malloy, K. Kernstine, R. E. Lenkinski, R. J. DeBerardinis,Metabolic heterogeneity in human lung tumors. Cell 164,681–694 (2016). 26853473 10.1016/j.cell.2015.12.034 PMC4752889

[13] K. D. Courtney, D. Bezwada, T. Mashimo, K. Pichumani, V. Vemireddy, A. M. Funk, J. Wimberly, S. S. McNeil, P. Kapur, Y. Lotan, V. Margulis, J. A. Cadeddu, I. Pedrosa, R. J. DeBerardinis, C. R. Malloy, R. M. Bachoo, E. A. Maher,Isotope tracing of human clear cell renal cell carcinomas demonstrates suppressed glucose oxidation in vivo. Cell Metab. 28,793–800.e2 (2018). 30146487 10.1016/j.cmet.2018.07.020 PMC6221993

[14] H. I. Wettersten, A. A. Hakimi, D. Morin, C. Bianchi, M. E. Johnstone, D. R. Donohoe, J. F. Trott, O. A. Aboud, S. Stirdivant, B. Neri, R. Wolfert, B. Stewart, R. Perego, J. J. Hsieh, R. H. Weiss,Grade-dependent metabolic reprogramming in kidney cancer revealed by combined proteomics and metabolomics analysis. Cancer Res. 75,2541–2552 (2015). 25952651 10.1158/0008-5472.CAN-14-1703 PMC4470795

[15] C. J. Ricketts, A. A. de Cubas, H. Fan, C. C. Smith, M. Lang, E. Reznik, R. Bowlby, E. A. Gibb, R. Akbani, R. Beroukhim, D. P. Bottaro, T. K. Choueiri, R. A. Gibbs, A. K. Godwin, S. Haake, A. A. Hakimi, E. P. Henske, J. J. Hsieh, T. H. Ho, R. S. Kanchi, B. Krishnan, D. J. Kwiatkowski, W. Lui, M. J. Merino, G. B. Mills, J. Myers, M. L. Nickerson, V. E. Reuter, L. S. Schmidt, C. S. Shelley, H. Shen, B. Shuch, S. Signoretti, R. Srinivasan, P. Tamboli, G. Thomas, B. G. Vincent, C. D. Vocke, D. A. Wheeler, L. Yang, W. Y. Kim, A. G. Robertson, P. T. Spellman, W. K. Rathmell, W. M. Linehan, S. J. Caesar-Johnson, J. A. Demchok, I. Felau, M. Kasapi, M. L. Ferguson, C. M. Hutter, H. J. Sofia, R. Tarnuzzer, Z. Wang, L. Yang, J. C. Zenklusen, J. (. J.). Zhang, S. Chudamani, J. Liu, L. Lolla, R. Naresh, T. Pihl, Q. Sun, Y. Wan, Y. Wu, J. Cho, T. DeFreitas, S. Frazer, N. Gehlenborg, G. Getz, D. I. Heiman, J. Kim, M. S. Lawrence, P. Lin, S. Meier, M. S. Noble, G. Saksena, D. Voet, H. Zhang, B. Bernard, N. Chambwe, V. Dhankani, T. Knijnenburg, R. Kramer, K. Leinonen, Y. Liu, M. Miller, S. Reynolds, I. Shmulevich, V. Thorsson, W. Zhang, R. Akbani, B. M. Broom, A. M. Hegde, Z. Ju, R. S. Kanchi, A. Korkut, J. Li, H. Liang, S. Ling, W. Liu, Y. Lu, G. B. Mills, K. S. Ng, A. Rao, M. Ryan, J. Wang, J. N. Weinstein, J. Zhang, A. Abeshouse, J. Armenia, D. Chakravarty, W. K. Chatila, I. de Bruijn, J. Gao, B. E. Gross, Z. J. Heins, R. Kundra, K. la, M. Ladanyi, A. Luna, M. G. Nissan, A. Ochoa, S. M. Phillips, E. Reznik, F. Sanchez-Vega, C. Sander, N. Schultz, R. Sheridan, S. O. Sumer, Y. Sun, B. S. Taylor, J. Wang, H. Zhang, P. Anur, M. Peto, P. Spellman, C. Benz, J. M. Stuart, C. K. Wong, C. Yau, D. N. Hayes, J. S. Parker, M. D. Wilkerson, A. Ally, M. Balasundaram, R. Bowlby, D. Brooks, R. Carlsen, E. Chuah, N. Dhalla, R. Holt, S. J. M. Jones, K. Kasaian, D. Lee, Y. Ma, M. A. Marra, M. Mayo, R. A. Moore, A. J. Mungall, K. Mungall, A. G. Robertson, S. Sadeghi, J. E. Schein, P. Sipahimalani, A. Tam, N. Thiessen, K. Tse, T. Wong, A. C. Berger, R. Beroukhim, A. D. Cherniack, C. Cibulskis, S. B. Gabriel, G. F. Gao, G. Ha, M. Meyerson, S. E. Schumacher, J. Shih, M. H. Kucherlapati, R. S. Kucherlapati, S. Baylin, L. Cope, L. Danilova, M. S. Bootwalla, P. H. Lai, D. T. Maglinte, D. J. van den Berg, D. J. Weisenberger, J. T. Auman, S. Balu, T. Bodenheimer, C. Fan, K. A. Hoadley, A. P. Hoyle, S. R. Jefferys, C. D. Jones, S. Meng, P. A. Mieczkowski, L. E. Mose, A. H. Perou, C. M. Perou, J. Roach, Y. Shi, J. V. Simons, T. Skelly, M. G. Soloway, D. Tan, U. Veluvolu, H. Fan, T. Hinoue, P. W. Laird, H. Shen, W. Zhou, M. Bellair, K. Chang, K. Covington, C. J. Creighton, H. Dinh, H. V. Doddapaneni, L. A. Donehower, J. Drummond, R. A. Gibbs, R. Glenn, W. Hale, Y. Han, J. Hu, V. Korchina, S. Lee, L. Lewis, W. Li, X. Liu, M. Morgan, D. Morton, D. Muzny, J. Santibanez, M. Sheth, E. Shinbrot, L. Wang, M. Wang, D. A. Wheeler, L. Xi, F. Zhao, J. Hess, E. L. Appelbaum, M. Bailey, M. G. Cordes, L. Ding, C. C. Fronick, L. A. Fulton, R. S. Fulton, C. Kandoth, E. R. Mardis, M. D. McLellan, C. A. Miller, H. K. Schmidt, R. K. Wilson, D. Crain, E. Curley, J. Gardner, K. Lau, D. Mallery, S. Morris, J. Paulauskis, R. Penny, C. Shelton, T. Shelton, M. Sherman, E. Thompson, P. Yena, J. Bowen, J. M. Gastier-Foster, M. Gerken, K. M. Leraas, T. M. Lichtenberg, N. C. Ramirez, L. Wise, E. Zmuda, N. Corcoran, T. Costello, C. Hovens, A. L. Carvalho, A. C. de Carvalho, J. H. Fregnani, A. Longatto-Filho, R. M. Reis, C. Scapulatempo-Neto, H. C. S. Silveira, D. O. Vidal, A. Burnette, J. Eschbacher, B. Hermes, A. Noss, R. Singh, M. L. Anderson, P. D. Castro, M. Ittmann, D. Huntsman, B. Kohl, X. le, R. Thorp, C. Andry, E. R. Duffy, V. Lyadov, O. Paklina, G. Setdikova, A. Shabunin, M. Tavobilov, C. McPherson, R. Warnick, R. Berkowitz, D. Cramer, C. Feltmate, N. Horowitz, A. Kibel, M. Muto, C. P. Raut, A. Malykh, J. S. Barnholtz-Sloan, W. Barrett, K. Devine, J. Fulop, Q. T. Ostrom, K. Shimmel, Y. Wolinsky, A. E. Sloan, A. de Rose, F. Giuliante, M. Goodman, B. Y. Karlan, C. H. Hagedorn, J. Eckman, J. Harr, J. Myers, K. Tucker, L. A. Zach, B. Deyarmin, H. Hu, L. Kvecher, C. Larson, R. J. Mural, S. Somiari, A. Vicha, T. Zelinka, J. Bennett, M. Iacocca, B. Rabeno, P. Swanson, M. Latour, L. Lacombe, B. Têtu, A. Bergeron, M. McGraw, S. M. Staugaitis, J. Chabot, H. Hibshoosh, A. Sepulveda, T. Su, T. Wang, O. Potapova, O. Voronina, L. Desjardins, O. Mariani, S. Roman-Roman, X. Sastre, M. H. Stern, F. Cheng, S. Signoretti, A. Berchuck, D. Bigner, E. Lipp, J. Marks, S. McCall, R. McLendon, A. Secord, A. Sharp, M. Behera, D. J. Brat, A. Chen, K. Delman, S. Force, F. Khuri, K. Magliocca, S. Maithel, J. J. Olson, T. Owonikoko, A. Pickens, S. Ramalingam, D. M. Shin, G. Sica, E. G. van Meir, H. Zhang, W. Eijckenboom, A. Gillis, E. Korpershoek, L. Looijenga, W. Oosterhuis, H. Stoop, K. E. van Kessel, E. C. Zwarthoff, C. Calatozzolo, L. Cuppini, S. Cuzzubbo, F. DiMeco, G. Finocchiaro, L. Mattei, A. Perin, B. Pollo, C. Chen, J. Houck, P. Lohavanichbutr, A. Hartmann, C. Stoehr, R. Stoehr, H. Taubert, S. Wach, B. Wullich, W. Kycler, D. Murawa, M. Wiznerowicz, K. Chung, W. J. Edenfield, J. Martin, E. Baudin, G. Bubley, R. Bueno, A. de Rienzo, W. G. Richards, S. Kalkanis, T. Mikkelsen, H. Noushmehr, L. Scarpace, N. Girard, M. Aymerich, E. Campo, E. Giné, A. L. Guillermo, N. van Bang, P. T. Hanh, B. D. Phu, Y. Tang, H. Colman, K. Evason, P. R. Dottino, J. A. Martignetti, H. Gabra, H. Juhl, T. Akeredolu, S. Stepa, D. Hoon, K. Ahn, K. J. Kang, F. Beuschlein, A. Breggia, M. Birrer, D. Bell, M. Borad, A. H. Bryce, E. Castle, V. Chandan, J. Cheville, J. A. Copland, M. Farnell, T. Flotte, N. Giama, T. Ho, M. Kendrick, J. P. Kocher, K. Kopp, C. Moser, D. Nagorney, D. O’Brien, B. P. O’Neill, T. Patel, G. Petersen, F. Que, M. Rivera, L. Roberts, R. Smallridge, T. Smyrk, M. Stanton, R. H. Thompson, M. Torbenson, J. D. Yang, L. Zhang, F. Brimo, J. A. Ajani, A. M. A. Gonzalez, C. Behrens, J. Bondaruk, R. Broaddus, B. Czerniak, B. Esmaeli, J. Fujimoto, J. Gershenwald, C. Guo, A. J. Lazar, C. Logothetis, F. Meric-Bernstam, C. Moran, L. Ramondetta, D. Rice, A. Sood, P. Tamboli, T. Thompson, P. Troncoso, A. Tsao, I. Wistuba, C. Carter, L. Haydu, P. Hersey, V. Jakrot, H. Kakavand, R. Kefford, K. Lee, G. Long, G. Mann, M. Quinn, R. Saw, R. Scolyer, K. Shannon, A. Spillane, Stretch, M. Synott, J. Thompson, J. Wilmott, H. al-Ahmadie, T. A. Chan, R. Ghossein, A. Gopalan, D. A. Levine, V. Reuter, S. Singer, B. Singh, N. V. Tien, T. Broudy, C. Mirsaidi, P. Nair, P. Drwiega, J. Miller, J. Smith, H. Zaren, J. W. Park, N. P. Hung, E. Kebebew, W. M. Linehan, A. R. Metwalli, K. Pacak, P. A. Pinto, M. Schiffman, L. S. Schmidt, C. D. Vocke, N. Wentzensen, R. Worrell, H. Yang, M. Moncrieff, C. Goparaju, J. Melamed, H. Pass, N. Botnariuc, I. Caraman, M. Cernat, I. Chemencedji, A. Clipca, S. Doruc, G. Gorincioi, S. Mura, M. Pirtac, I. Stancul, D. Tcaciuc, M. Albert, I. Alexopoulou, A. Arnaout, J. Bartlett, J. Engel, S. Gilbert, J. Parfitt, H. Sekhon, G. Thomas, D. M. Rassl, R. C. Rintoul, C. Bifulco, R. Tamakawa, W. Urba, N. Hayward, H. Timmers, A. Antenucci, F. Facciolo, G. Grazi, M. Marino, R. Merola, R. de Krijger, A. P. Gimenez-Roqueplo, A. Piché, S. Chevalier, G. McKercher, K. Birsoy, G. Barnett, C. Brewer, C. Farver, T. Naska, N. A. Pennell, D. Raymond, C. Schilero, K. Smolenski, F. Williams, C. Morrison, J. A. Borgia, M. J. Liptay, M. Pool, C. W. Seder, K. Junker, L. Omberg, M. Dinkin, G. Manikhas, D. Alvaro, M. C. Bragazzi, V. Cardinale, G. Carpino, E. Gaudio, D. Chesla, S. Cottingham, M. Dubina, F. Moiseenko, R. Dhanasekaran, K. F. Becker, K. P. Janssen, J. Slotta-Huspenina, M. H. Abdel-Rahman, D. Aziz, S. Bell, C. M. Cebulla, A. Davis, R. Duell, J. B. Elder, J. Hilty, B. Kumar, J. Lang, N. L. Lehman, R. Mandt, P. Nguyen, R. Pilarski, K. Rai, L. Schoenfield, K. Senecal, P. Wakely, P. Hansen, R. Lechan, J. Powers, A. Tischler, W. E. Grizzle, K. C. Sexton, A. Kastl, J. Henderson, S. Porten, J. Waldmann, M. Fassnacht, S. L. Asa, D. Schadendorf, M. Couce, M. Graefen, H. Huland, G. Sauter, T. Schlomm, R. Simon, P. Tennstedt, O. Olabode, M. Nelson, O. Bathe, P. R. Carroll, J. M. Chan, P. Disaia, P. Glenn, R. K. Kelley, C. N. Landen, J. Phillips, M. Prados, J. Simko, K. Smith-McCune, S. VandenBerg, K. Roggin, A. Fehrenbach, A. Kendler, S. Sifri, R. Steele, A. Jimeno, F. Carey, I. Forgie, M. Mannelli, M. Carney, B. Hernandez, B. Campos, C. Herold-Mende, C. Jungk, A. Unterberg, A. von Deimling, A. Bossler, J. Galbraith, L. Jacobus, M. Knudson, T. Knutson, D. Ma, M. Milhem, R. Sigmund, A. K. Godwin, R. Madan, H. G. Rosenthal, C. Adebamowo, S. N. Adebamowo, A. Boussioutas, D. Beer, T. Giordano, A. M. Mes-Masson, F. Saad, T. Bocklage, L. Landrum, R. Mannel, K. Moore, K. Moxley, R. Postier, J. Walker, R. Zuna, M. Feldman, F. Valdivieso, R. Dhir, J. Luketich, E. M. M. Pinero, M. Quintero-Aguilo, C. G. Carlotti Jr., J. S. dos Santos, R. Kemp, A. Sankarankuty, D. Tirapelli, J. Catto, K. Agnew, E. Swisher, J. Creaney, B. Robinson, C. S. Shelley, E. M. Godwin, S. Kendall, C. Shipman, C. Bradford, T. Carey, A. Haddad, J. Moyer, L. Peterson, M. Prince, L. Rozek, G. Wolf, R. Bowman, K. M. Fong, I. Yang, R. Korst, W. K. Rathmell, J. L. Fantacone-Campbell, J. A. Hooke, A. J. Kovatich, C. D. Shriver, J. DiPersio, B. Drake, R. Govindan, S. Heath, T. Ley, B. van Tine, P. Westervelt, M. A. Rubin, J. I. Lee, N. D. Aredes, A. Mariamidze,The Cancer Genome Atlas comprehensive molecular characterization of renal cell carcinoma. Cell Rep. 23,313–326.e5 (2018). 29617669 10.1016/j.celrep.2018.03.075 PMC6075733

[16] B. Li, B. Qiu, D. S. M. Lee, Z. E. Walton, J. D. Ochocki, L. K. Mathew, A. Mancuso, T. P. F. Gade, B. Keith, I. Nissim, M. C. Simon,Fructose-1,6-bisphosphatase opposes renal carcinoma progression. Nature 513,251–255 (2014). 25043030 10.1038/nature13557 PMC4162811

[17] A. A. Hakimi, E. Reznik, C. H. Lee, C. J. Creighton, A. R. Brannon, A. Luna, B. A. Aksoy, E. M. Liu, R. Shen, W. Lee, Y. Chen, S. M. Stirdivant, P. Russo, Y. B. Chen, S. K. Tickoo, V. E. Reuter, E. H. Cheng, C. Sander, J. J. Hsieh,An integrated metabolic atlas of clear cell renal cell carcinoma. Cancer Cell 29,104–116 (2016). 26766592 10.1016/j.ccell.2015.12.004 PMC4809063

[18] A. R. Mullen, W. W. Wheaton, E. S. Jin, P. H. Chen, L. B. Sullivan, T. Cheng, Y. Yang, W. M. Linehan, N. S. Chandel, R. DeBerardinis,Reductive carboxylation supports growth in tumour cells with defective mitochondria. Nature 481,385–388 (2011). 22101431 10.1038/nature10642 PMC3262117

[19] C. M. Metallo, P. A. Gameiro, E. L. Bell, K. R. Mattaini, J. Yang, K. Hiller, C. M. Jewell, Z. R. Johnson, D. J. Irvine, L. Guarente, J. K. Kelleher, M. G. Vander Heiden, O. Iliopoulos, G. Stephanopoulos,Reductive glutamine metabolism by IDH1 mediates lipogenesis under hypoxia. Nature 481,380–384 (2011). 22101433 10.1038/nature10602 PMC3710581

[20] P. A. Gameiro, J. Yang, A. M. Metelo, R. Pérez-Carro, R. Baker, Z. Wang, A. Arreola, W. K. Rathmell, A. Olumi, P. López-Larrubia, G. Stephanopoulos, O. Iliopoulos,In vivo HIF-mediated reductive carboxylation is regulated by citrate levels and sensitizes VHL-deficient cells to glutamine deprivation. Cell Metab. 17,372–385 (2013). 23473032 10.1016/j.cmet.2013.02.002 PMC4003458

[21] F. Meric-Bernstam, N. M. Tannir, J. W. Mier, A. DeMichele, M. L. Telli, A. C. Fan, P. N. Munster, R. D. Carvajal, K. W. Orford, M. K. Bennett, O. Iliopoulos, T. K. Owonikoko, M. R. Patel, R. McKay, J. R. Infante, M. H. Voss, J. J. Harding,Phase 1 study of CB-839, a small molecule inhibitor of glutaminase (GLS), alone and in combination with everolimus (E) in patients (pts) with renal cell cancer (RCC). J. Clin. Oncol. 34,4568 (2016).

[22] J. J. Harding, M. Telli, P. Munster, M. H. Voss, J. R. Infante, A. DeMichele, M. Dunphy, M. H. le, C. Molineaux, K. Orford, F. Parlati, S. H. Whiting, M. K. Bennett, N. M. Tannir, F. Meric-Bernstam,A phase I dose-escalation and expansion study of telaglenastat in patients with advanced or metastatic solid tumors. Clin. Cancer Res. 27,4994–5003 (2021). 34285061 10.1158/1078-0432.CCR-21-1204 PMC9401498

[23] N. M. Tannir, N. Agarwal, C. Porta, N. J. Lawrence, R. J. Motzer, R. J. Lee, R. K. Jain, N. B. Davis, L. J. Appleman, O. B. Goodman, W. M. Stadler, S. G. Gandhi, D. M. Geynisman, R. Iacovelli, B. Mellado, R. A. Figlin, T. Powles, L. V. Akella, K. W. Orford, B. Escudier,CANTATA: Primary analysis of a global, randomized, placebo (Pbo)-controlled, double-blind trial of telaglenastat (CB-839) + cabozantinib versus Pbo + cabozantinib in advanced/metastatic renal cell carcinoma (mRCC) patients (pts) who progressed on immune checkpoint inhibitor (ICI) or anti-angiogenic therapies. J. Clin. Oncol. 39,4501 (2021).

[24] L. Yang, S. Venneti, D. Nagrath,Glutaminolysis: A hallmark of cancer metabolism. Annu. Rev. Biomed. Eng. 19,163–194 (2017). 28301735 10.1146/annurev-bioeng-071516-044546

[25] R. D. Leone, L. Zhao, J. M. Englert, I.-M. Sun, M.-H. Oh, I.-H. Sun, M. L. Arwood, I. A. Bettencourt, C. H. Patel, J. Wen, A. Tam, R. L. Blosser, E. Prchalova, J. Alt, R. Rais, B. S. Slusher, J. D. Powell,Glutamine blockade induces divergent metabolic programs to overcome tumor immune evasion. Science 366,1013–1021 (2019). 31699883 10.1126/science.aav2588 PMC7023461

[26] R. J. DeBerardinis, A. Mancuso, E. Daikhin, I. Nissim, M. Yudkoff, S. Wehrli, C. B. Thompson,Beyond aerobic glycolysis: Transformed cells can engage in glutamine metabolism that exceeds the requirement for protein and nucleotide synthesis. Proc. Natl. Acad. Sci. U.S.A. 104,19345–19350 (2007). 18032601 10.1073/pnas.0709747104 PMC2148292

[27] I. Marin-Valencia, C. Yang, T. Mashimo, S. Cho, H. Baek, X. L. Yang, K. N. Rajagopalan, M. Maddie, V. Vemireddy, Z. Zhao, L. Cai, L. Good, B. P. Tu, K. J. Hatanpaa, B. E. Mickey, J. M. Matés, J. M. Pascual, E. A. Maher, C. R. Malloy, R. J. DeBerardinis, R. M. Bachoo,Analysis of tumor metabolism reveals mitochondrial glucose oxidation in genetically diverse human glioblastomas in the mouse brain in vivo. Cell Metab. 15,827–837 (2012). 22682223 10.1016/j.cmet.2012.05.001 PMC3372870

[28] S. M. Davidson, T. Papagiannakopoulos, B. A. Olenchock, J. E. Heyman, M. A. Keibler, A. Luengo, M. R. Bauer, A. K. Jha, J. P. O’Brien, K. A. Pierce, D. Y. Gui, L. B. Sullivan, T. M. Wasylenko, L. Subbaraj, C. R. Chin, G. Stephanopolous, B. T. Mott, T. Jacks, C. B. Clish, M. G. Vander Heiden,Environment impacts the metabolic dependencies of Ras-driven non-small cell lung cancer. Cell Metab. 23,517–528 (2016). 26853747 10.1016/j.cmet.2016.01.007 PMC4785096

[29] S. Sivanand, S. Peña-Llopis, H. Zhao, B. Kucejova, P. Spence, A. Pavia-Jimenez, T. Yamasaki, D. J. McBride, J. Gillen, N. C. Wolff, L. Morlock, Y. Lotan, G. V. Raj, A. Sagalowsky, V. Margulis, J. A. Cadeddu, M. T. Ross, D. R. Bentley, W. Kabbani, X.-J. Xie, P. Kapur, N. S. Williams, J. Brugarolas,A validated tumorgraft model reveals activity of dovitinib against renal cell carcinoma. Sci. Transl. Med. 4,137ra75 (2012). 10.1126/scitranslmed.3003643 PMC3570965 22674553

[30] R. Elias, V. T. Tcheuyap, A. K. Kaushik, N. Singla, M. Gao, O. Reig Torras, A. Christie, A. Mulgaonkar, L. Woolford, C. Stevens, K. P. Kettimuthu, A. Pavia-Jimenez, L. K. Boroughs, A. Joyce, M. Dakanali, H. Notgrass, V. Margulis, J. A. Cadeddu, I. Pedrosa, N. S. Williams, X. Sun, R. J. DeBerardinis, O. K. Öz, H. Zhong, S. Seshagiri, Z. Modrusan, B. L. Cantarel, P. Kapur, J. Brugarolas,A renal cell carcinoma tumorgraft platform to advance precision medicine. Cell Rep. 37,110055 (2021). 34818533 10.1016/j.celrep.2021.110055 PMC8762721

[31] A. Pavia-Jimenez, V. T. Tcheuyap, J. Brugarolas,Establishing a human renal cell carcinoma tumorgraft platform for preclinical drug testing. Nat. Protoc. 9,1848–1859 (2014). 25010905 10.1038/nprot.2014.108 PMC4314943

[32] R. Gupta, F. Ionescu, V. Jindal, J. Khoury, N. I. Anusim, I. A. Jaiyesimi,Survival outcomes of sarcomatoid renal cell cancer (sRCC) compared to clear cell renal cell cancer (ccRCC): An analysis of SEER data. J. Clin. Oncol. 38,e17101 (2020).

[33] Y. Zhang, D. Udayakumar, L. Cai, Z. Hu, P. Kapur, E. Y. Kho, A. Pavía-Jiménez, M. Fulkerson, A. D. de Leon, Q. Yuan, I. E. Dimitrov, T. Yokoo, J. Ye, M. A. Mitsche, H. Kim, J. G. McDonald, Y. Xi, A. J. Madhuranthakam, D. K. Dwivedi, R. E. Lenkinski, J. A. Cadeddu, V. Margulis, J. Brugarolas, R. J. DeBerardinis, I. Pedrosa,Addressing metabolic heterogeneity in clear cell renal cell carcinoma with quantitative Dixon MRI. JCI Insight 2,e94278 (2017). 28768909 10.1172/jci.insight.94278 PMC5543910

[34] K. N. Rajagopalan, R. A. Egnatchik, M. A. Calvaruso, A. T. Wasti, M. S. Padanad, L. K. Boroughs, B. Ko, C. T. Hensley, M. Acar, Z. Hu, L. Jiang, J. M. Pascual, P. P. Scaglioni, R. J. DeBerardinis,Metabolic plasticity maintains proliferation in pyruvate dehydrogenase deficient cells. Cancer Metab. 3,7 (2015). 26137220 10.1186/s40170-015-0134-4 PMC4487196

[35] S. M. Fendt, E. L. Bell, M. A. Keibler, B. A. Olenchock, J. R. Mayers, T. M. Wasylenko, N. I. Vokes, L. Guarente, M. G. V. Heiden, G. Stephanopoulos,Reductive glutamine metabolism is a function of the α-ketoglutarate to citrate ratio in cells. Nat. Commun. 4,2236 (2013). 23900562 10.1038/ncomms3236 PMC3934748

[36] M. O. Yuneva, T. W. M. Fan, T. D. Allen, R. M. Higashi, D. V. Ferraris, T. Tsukamoto, J. M. Matés, F. J. Alonso, C. Wang, Y. Seo, X. Chen, J. M. Bishop,The metabolic profile of tumors depends on both the responsible genetic lesion and tissue type. Cell Metab. 15,157–170 (2012). 22326218 10.1016/j.cmet.2011.12.015 PMC3282107

[37] J. R. Mayers, M. E. Torrence, L. V. Danai, T. Papagiannakopoulos, S. M. Davidson, M. R. Bauer, A. N. Lau, B. W. Ji, P. D. Dixit, A. M. Hosios, A. Muir, C. R. Chin, E. Freinkman, T. Jacks, B. M. Wolpin, D. Vitkup, M. G. Vander Heiden,Tissue of origin dictates branched-chain amino acid metabolism in mutant Kras-driven cancers. Science 353,1161–1165 (2016). 27609895 10.1126/science.aaf5171 PMC5245791

[38] E. H. Shim, C. B. Livi, D. Rakheja, J. Tan, D. Benson, V. Parekh, E. Y. Kho, A. P. Ghosh, R. Kirkman, S. Velu, S. Dutta, B. Chenna, S. L. Rea, R. J. Mishur, Q. Li, T. L. Johnson-Pais, L. Guo, S. Bae, S. Wei, K. Block, S. Sudarshan,L-2-hydroxyglutarate: An epigenetic modifier and putative oncometabolite in renal cancer. Cancer Discov. 4,1290–1298 (2014). 25182153 10.1158/2159-8290.CD-13-0696 PMC4286872

[39] A. S. Cohen, J. Grudzinski, G. T. Smith, T. E. Peterson, J. G. Whisenant, T. L. Hickman, K. K. Ciombor, D. Cardin, C. Eng, L. W. Goff, S. Das, R. J. Coffey, J. D. Berlin, H. C. Manning,First-in-human PET imaging and estimated radiation dosimetry of L-[5-^11^C]-glutamine in patients with metastatic colorectal cancer. J. Nucl. Med. 63,36–43 (2022). 33931465 10.2967/jnumed.120.261594 PMC8717201

[40] S. Tommasini-Ghelfi, K. Murnan, F. M. Kouri, A. S. Mahajan, J. L. May, A. H. Stegh,Cancer-associated mutation and beyond: The emerging biology of isocitrate dehydrogenases in human disease. Sci. Adv. 5,eaaw4543 (2019). 31131326 10.1126/sciadv.aaw4543 PMC6530995

[41] A. Mendez-Lucas, W. Lin, P. C. Driscoll, N. Legrave, L. Novellasdemunt, C. Xie, M. Charles, Z. Wilson, N. P. Jones, S. Rayport, M. Rodríguez-Justo, V. Li, J. I. MacRae, N. Hay, X. Chen, M. Yuneva,Identifying strategies to target the metabolic flexibility of tumours. Nat. Metab. 2,335–350 (2020). 32694609 10.1038/s42255-020-0195-8 PMC7436715

[42] B. I. Reinfeld, M. Z. Madden, M. M. Wolf, A. Chytil, J. E. Bader, A. R. Patterson, A. Sugiura, A. S. Cohen, A. Ali, B. T. do, A. Muir, C. A. Lewis, R. A. Hongo, K. L. Young, R. E. Brown, V. M. Todd, T. Huffstater, A. Abraham, R. T. O’Neil, M. H. Wilson, F. Xin, M. N. Tantawy, W. D. Merryman, R. W. Johnson, C. S. Williams, E. F. Mason, F. M. Mason, K. E. Beckermann, M. G. Vander Heiden, H. C. Manning, J. C. Rathmell, W. K. Rathmell,Cell-programmed nutrient partitioning in the tumour microenvironment. Nature 593,282–288 (2021). 33828302 10.1038/s41586-021-03442-1 PMC8122068

[43] A. Vaziri-Gohar, J. Cassel, F. S. Mohammed, M. Zarei, J. J. Hue, O. Hajihassani, H. J. Graor, Y. V. V. Srikanth, S. A. Karim, A. Abbas, E. Prendergast, V. Chen, E. S. Katayama, K. Dukleska, I. Khokhar, A. Andren, L. Zhang, C. Wu, B. Erokwu, C. A. Flask, M. Zarei, R. Wang, L. D. Rothermel, A. M. P. Romani, J. Bowers, R. Getts, C. Tatsuoka, J. P. Morton, I. Bederman, H. Brunengraber, C. A. Lyssiotis, J. M. Salvino, J. R. Brody, J. M. Winter,Limited nutrient availability in the tumor microenvironment renders pancreatic tumors sensitive to allosteric IDH1 inhibitors. Nat. Cancer 3,852–865 (2022). 35681100 10.1038/s43018-022-00393-y PMC9325670

[44] L. Jiang, A. A. Shestov, P. Swain, C. Yang, S. J. Parker, Q. A. Wang, L. S. Terada, N. D. Adams, M. T. McCabe, B. Pietrak, S. Schmidt, C. M. Metallo, B. P. Dranka, B. Schwartz, R. J. DeBerardinis,Reductive carboxylation supports redox homeostasis during anchorage-independent growth. Nature 532,255–258 (2016). 27049945 10.1038/nature17393 PMC4860952

[45] Z. Konteatis, E. Artin, B. Nicolay, K. Straley, A. K. Padyana, L. Jin, Y. Chen, R. Narayaraswamy, S. Tong, F. Wang, D. Zhou, D. Cui, Z. Cai, Z. Luo, C. Fang, H. Tang, X. Lv, R. Nagaraja, H. Yang, S. S. M. Su, Z. Sui, L. Dang, K. Yen, J. Popovici-Muller, P. Codega, C. Campos, I. K. Mellinghoff, S. A. Biller,Vorasidenib (AG-881): A first-in-class, brain-penetrant dual inhibitor of mutant IDH1 and 2 for treatment of glioma. ACS Med. Chem. Lett. 11,101–107 (2020). 32071674 10.1021/acsmedchemlett.9b00509 PMC7025383

[46] M. L. Schulte, A. Fu, P. Zhao, J. Li, L. Geng, S. T. Smith, J. Kondo, R. J. Coffey, M. O. Johnson, J. C. Rathmell, J. T. Sharick, M. C. Skala, J. A. Smith, J. Berlin, M. K. Washington, M. L. Nickels, H. C. Manning,Pharmacological blockade of ASCT2-dependent glutamine transport leads to antitumor efficacy in preclinical models. Nat. Med. 24,194–202 (2018). 29334372 10.1038/nm.4464 PMC5803339

[47] E. H. Shroff, L. S. Eberlin, V. M. Dang, A. M. Gouw, M. Gabay, S. J. Adam, D. I. Bellovin, P. T. Tran, W. M. Philbrick, A. Garcia-Ocana, S. C. Casey, Y. Li, C. V. Dang, R. N. Zare, D. W. Felsher,MYC oncogene overexpression drives renal cell carcinoma in a mouse model through glutamine metabolism. Proc. Natl. Acad. Sci. U.S.A. 112,6539–6544 (2015). 25964345 10.1073/pnas.1507228112 PMC4450371

[48] M. I. Gross, S. D. Demo, J. B. Dennison, L. Chen, T. Chernov-Rogan, B. Goyal, J. R. Janes, G. J. Laidig, E. R. Lewis, J. Li, A. L. MacKinnon, F. Parlati, M. L. M. Rodriguez, P. J. Shwonek, E. B. Sjogren, T. F. Stanton, T. Wang, J. Yang, F. Zhao, M. K. Bennett,Antitumor activity of the glutaminase inhibitor CB-839 in triple-negative breast cancer. Mol. Cancer Ther. 13,890–901 (2014). 24523301 10.1158/1535-7163.MCT-13-0870

[49] R. Romero, V. I. Sayin, S. M. Davidson, M. R. Bauer, S. X. Singh, S. E. LeBoeuf, T. R. Karakousi, D. C. Ellis, A. Bhutkar, F. J. Sánchez-Rivera, L. Subbaraj, B. Martinez, R. T. Bronson, J. R. Prigge, E. E. Schmidt, C. J. Thomas, C. Goparaju, A. Davies, I. Dolgalev, A. Heguy, V. Allaj, J. T. Poirier, A. L. Moreira, C. M. Rudin, H. I. Pass, M. G. Vander Heiden, T. Jacks, T. Papagiannakopoulos,Keap1 loss promotes Kras-driven lung cancer and results in dependence on glutaminolysis. Nat. Med. 23,1362–1368 (2017). 28967920 10.1038/nm.4407 PMC5677540

[50] H. Ding, Z. Chen, K. Wu, S. M. Huang, W. L. Wu, S. E. LeBoeuf, R. G. Pillai, J. D. Rabinowitz, T. Papagiannakopoulos,Activation of the NRF2 antioxidant program sensitizes tumors to G6PD inhibition. Sci. Adv. 7,eabk1023 (2021). 34788087 10.1126/sciadv.abk1023 PMC8598006

[51] N. M. Tannir, A. C. Fan, R. J. Lee, B. C. Carthon, O. Iliopoulos, J. W. Mier, M. R. Patel, F. Meric-Bernstam, A. DeMichele, M. H. Voss, J. J. Harding, R. Srinivasan, G. Shapiro, M. L. Telli, P. N. Munster, R. D. Carvajal, Y. Jenkins, S. H. Whiting, J. C. Bendell, T. M. Bauer,Phase 1 study of glutaminase (GLS) inhibitor CB-839 combined with either everolimus (E) or cabozantinib (Cabo) in patients (pts) with clear cell (cc) and papillary (pap) metastatic renal cell cancer (mRCC). J. Clin. Oncol. 36,603–603 (2018).

[52] G. B. Magill, W. P. L. Myers, H. C. Reilly, R. C. Putnam, J. W. Magill, M. P. Sykes, G. C. Escher, D. A. Karnofsky, J. H. Burchenal,Pharmacological and initial therapeutic observations on 6-diazo-5-oxo-1-norleucine (DON) in human neoplastic disease. Cancer 10,1138–1150 (1957). 13489662 10.1002/1097-0142(195711/12)10:6<1138::aid-cncr2820100608>3.0.co;2-k

[53] Veterans Administration Cancer Chemotherapy Study Group, S. Krantz, S. Rivers, R. W. Dwight, H. F. Corbus, J. Wolf, I. Green, P. W. Spear, L. T. Imperato, S. Lobe, R. M. Whittington, J. M. Rumball, A. Marquez, C. Cables, A. I. Chernoff, D. K. Misra, R. D. Sullivan, E. Miller, F. S. Dietrich, G. I. Plitman, H. P. Close, S. M. Cracken, A. S. Glushien, D. L. Rucknagel, C. C. Li, D. Kodlin,A CLINICAL study of the comparative effect of nitrogen mustard and DON in patients with bronchogenic carcinoma, Hodgkin's disease, lymphosarcoma, and melanoma. J. Natl. Cancer Inst. 22,433–439 (1959). 13631504

[54] K. M. Lemberg, J. J. Vornov, R. Rais, B. S. Slusher,We're not "DON" yet: Optimal dosing and prodrug delivery of 6-diazo-5-oxo-l-norleucine. Mol. Cancer Ther. 17,1824–1832 (2018). 30181331 10.1158/1535-7163.MCT-17-1148 PMC6130910

[55] M. H. Oh, I. H. Sun, L. Zhao, R. D. Leone, I. M. Sun, W. Xu, S. L. Collins, A. J. Tam, R. L. Blosser, C. H. Patel, J. M. Englert, M. L. Arwood, J. Wen, Y. Chan-Li, L. Tenora, P. Majer, R. Rais, B. S. Slusher, M. R. Horton, J. D. Powell,Targeting glutamine metabolism enhances tumor-specific immunity by modulating suppressive myeloid cells. J. Clin. Invest. 130,3865–3884 (2020). 32324593 10.1172/JCI131859 PMC7324212

[56] A. R. Hanaford, J. Alt, R. Rais, S. Z. Wang, H. Kaur, D. L. J. Thorek, C. G. Eberhart, B. S. Slusher, A. M. Martin, E. H. Raabe,Orally bioavailable glutamine antagonist prodrug JHU-083 penetrates mouse brain and suppresses the growth of MYC-driven medulloblastoma. Transl. Oncol. 12,1314–1322 (2019). 31340195 10.1016/j.tranon.2019.05.013 PMC6657308

[57] N. H. Chakiryan, D. D. Jiang, K. A. Gillis, E. Green, A. Hajiran, L. Hugar, L. Zemp, J. Zhang, R. K. Jain, J. Chahoud, P. E. Spiess, W. Sexton, S. M. Gilbert, B. J. Manley,Real-world survival outcomes associated with first-line immunotherapy, targeted therapy, and combination therapy for metastatic clear cell renal cell carcinoma. JAMA Netw. Open 4,e2111329 (2021). 34032854 10.1001/jamanetworkopen.2021.11329 PMC8150693

[58] B. I. Rini, E. R. Plimack, V. Stus, T. Waddell, R. Gafanov, F. Pouliot, D. Nosov, B. Melichar, D. Soulieres, D. Borchiellini, I. O. Vynnychenko, R. S. McDermott, S. J. Azevedo, S. Tamada, A. Kryzhanivska, C. Li, J. E. Burgents, L. R. Molife, J. Bedke, T. Powles,Pembrolizumab (pembro) plus axitinib (axi) versus sunitinib as first-line therapy for advanced clear cell renal cell carcinoma (ccRCC): Results from 42-month follow-up of KEYNOTE-426. J. Clin. Oncol. 39,4500–4500 (2021).

[59] A. S. Yamashita, M. da Costa Rosa, V. Stumpo, R. Rais, B. S. Slusher, G. J. Riggins,The glutamine antagonist prodrug JHU-083 slows malignant glioma growth and disrupts mTOR signaling. Neurooncol Adv. 3, (2021). 10.1093/noajnl/vdaa149 PMC7920530 33681764

[60] A. R. Mullen, Z. Hu, X. Shi, L. Jiang, L. K. Boroughs, Z. Kovacs, R. Boriack, D. Rakheja, L. B. Sullivan, W. M. Linehan, N. S. Chandel, R. J. DeBerardinis,Oxidation of alpha-ketoglutarate is required for reductive carboxylation in cancer cells with mitochondrial defects. Cell Rep. 7,1679–1690 (2014). 24857658 10.1016/j.celrep.2014.04.037 PMC4057960

[61] J. Chong, D. S. Wishart, J. Xia,Using MetaboAnalyst 4.0 for comprehensive and integrative metabolomics data analysis. Curr. Protoc. Bioinformatics 68,e86 (2019). 31756036 10.1002/cpbi.86

[62] C. R. Bartman, Y. Shen, W. D. Lee, T. TeSlaa, C. S. R. Jankowski, L. Wang, L. Yang, A. Roichman, V. Bhatt, T. Lan, Z. Hu, X. Xing, W. Lu, J. Y. Guo, J. D. Rabinowitz, Slow TCA flux implies low ATP production in tumors. bioRxiv 2021.10.04.463108 [Preprint]. 4 October 2021. 10.1101/2021.10.04.463108.

[63] A. Tasdogan, B. Faubert, V. Ramesh, J. M. Ubellacker, B. Shen, A. Solmonson, M. M. Murphy, Z. Gu, W. Gu, M. Martin, S. Y. Kasitinon, T. Vandergriff, T. P. Mathews, Z. Zhao, D. Schadendorf, R. J. DeBerardinis, S. J. Morrison,Metabolic heterogeneity confers differences in melanoma metastatic potential. Nature 577,115–120 (2020). 31853067 10.1038/s41586-019-1847-2 PMC6930341

[64] P. Heinrich, C. Kohler, L. Ellmann, P. Kuerner, R. Spang, P. J. Oefner, K. Dettmer,Correcting for natural isotope abundance and tracer impurity in MS-, MS/MS- and high-resolution-multiple-tracer-data from stable isotope labeling experiments with IsoCorrectoR. Sci. Rep. 8,17910 (2018). 30559398 10.1038/s41598-018-36293-4 PMC6297158

[65] E. A. Struys, E. E. Jansen, N. M. Verhoeven, C. Jakobs,Measurement of urinary d- and l-2-hydroxyglutarate enantiomers by stable-isotope-dilution liquid chromatography-tandem mass spectrometry after derivatization with diacetyl-l-tartaric anhydride. Clin. Chem. 50,1391–1395 (2004). 15166110 10.1373/clinchem.2004.033399

[66] V. Ramaswamy, J. W. Hooker, R. S. Withers, R. E. Nast, W. W. Brey, A. S. Edison,Development of a ^13^C-optimized 1.5-mm high temperature superconducting NMR probe. J. Magn. Reson. 235,58–65 (2013). 23969086 10.1016/j.jmr.2013.07.012 PMC3785096

[67] N. E. Sanjana, O. Shalem, F. Zhang,Improved vectors and genome-wide libraries for CRISPR screening. Nat. Methods 11,783–784 (2014). 25075903 10.1038/nmeth.3047 PMC4486245

