# Supplementary material for: In vivo characterization of glutamine metabolism identifies therapeutic targets in clear cell renal cell carcinoma
Source: Sci Adv. 2022 Dec 16;8(50):eabp8293. doi: 10.1126/sciadv.abp8293 (PMC9757752; doi:10.1126/sciadv.abp8293)
Supplement: Supplementary file 1 — Supplementary Text Figs. S1 to S9 Table S1 [file sciadv.abp8293_sm.pdf]

Science Advances
AAAS

Supplementary Materials for

In vivo characterization of glutamine metabolism identifies therapeutic targets in clear cell renal cell carcinoma

Akash K. Kaushik *et al.*

Corresponding author: Ralph J. DeBerardinis, ralph.deberardinis@utsouthwestern.edu

Sci. Adv. **8**, eabp8293 (2022)
DOI: 10.1126/sciadv.abp8293

The PDF file includes:

Supplementary Text
Figs. S1 to S9
Table S1
Legends for data S1 and S2

Other Supplementary Material for this manuscript includes the following:

Data S1 and S2

A detailed explanation of ^{13}C-NMR data

Among all *VHL*-mutant tumors examined in this study, XP955 tumors had the highest labeling in aspartate and malate from the reductive pathway, with M+3 labeling in these metabolites exceeding M+5 labeled citrate (Fig. S3G). This is an unusual pattern because citrate is upstream of aspartate and malate in this pathway and would presumably have equivalent or higher labeling (Fig. 2B). To provide further detail to the tracer analysis, we used NMR to examine the ^{13}C positional enrichment in metabolites after infusion with [U^{13}C]glutamine. Glutamine oxidation produces uniformly-labeled malate on the first turn of the TCA cycle, so spin-coupling generates complex NMR multiplets at malate C2 and C3. Specifically, oxidative metabolism should produce quartets (i.e., doublets of doublets), reflecting labeling in C1-3 observable at the C2 chemical shift and C2-4 observable at the C3 chemical shift. Reductive metabolism generates citrate labeled at all positions except C6, and cleavage by ATP citrate lyase generates OAA and malate labeled in C2-4 (Fig. S4B). Equilibration with fumarate, a symmetric metabolite, ultimately results in malate labeled in either C1-3 or C2-4 in equal measures. Therefore, reductive metabolism also produces multiplets at malate C2 and C3, including quartets. However, unlike the uniformly labeled malate from the oxidative pathway, malate from the reductive pathway lacks ^{13}C on either C1 or C4. This produces malate C1-3 and C2-4 isotopomers that give rise to doublets at the C2 and C3 positions in addition to contributions from the quartets (i.e., at C2, the doublets reflect malate molecules containing ^{13}C at C2 and C3

but not C1; and at C3, malate molecules containing ^{13}C at C2 and C3 but not C4). These doublets should be similar in size at steady state.

NMR spectra of metabolites extracted from [U-^{13}C]glutamine-infused tumors revealed complex C2 and C3 malate multiplets, but little malate signal was detected in spectra from the kidney (Fig. S4C,D). In tumor metabolites, quartets and 2-3 doublets were prominent at both C2 (C2D23) and C3 (C3D23), consistent with reductive labeling in agreement with the mass spectrometry data. To more directly assess the contribution of oxidative metabolism, we examined 1-2 and 3-4 doublets in malate; these patterns arise on the second oxidative turn of the cycle and were barely detectable (Fig. S4B-D). Some ^{13}C label was also detected at resonances for C2/C4 in tumor-derived citrate (Fig. S4C, E), but the signal-to-noise ratio was much lower than for malate, again consistent with the low citrate labeling observed by mass spectrometry. Altogether, these data indicate prominent reductive and suppressed oxidative labeling from glutamine in this ccRCC model. The low citrate enrichment may reflect contributions of an unlabeled citrate pool arising from sources other than glutamine. However, glutamine-derived citrate contributes to other TCA cycle intermediates, resulting in relatively high enrichment in these metabolites.

Figure S1

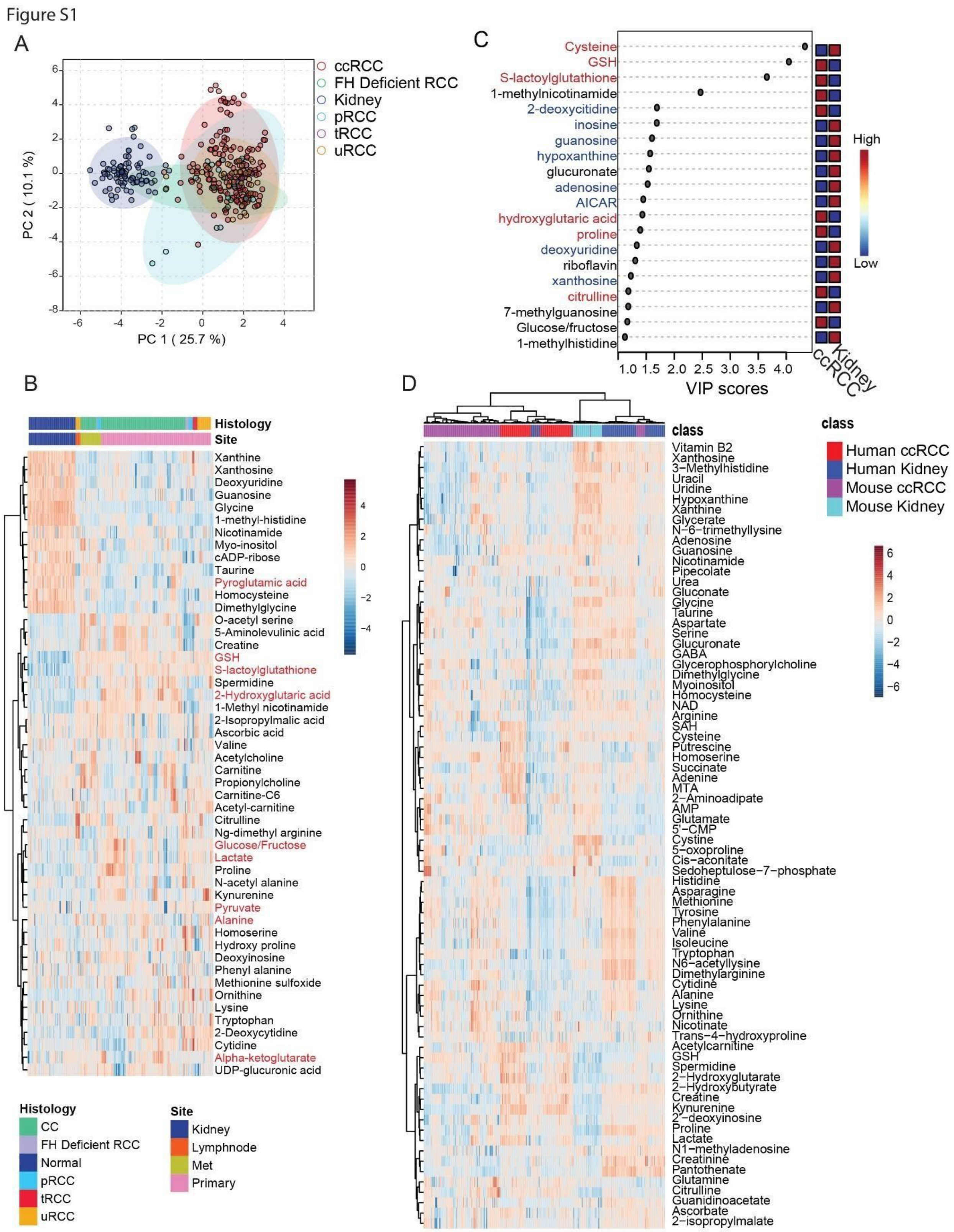
Figure S1
A
ccRCC
FH Deficient RCC
Kidney
pRCC
tRCC
uRCC
PC 2 (10.1 %)
PC 1 (25.7 %)
C
Cysteine
GSH
S-lactoylglutathione
1-methylnicotinamide
2-deoxycitidine
inosine
guanosine
hypoxanthine
glucuronate
adenosine
AICAR
hydroxyglutaric acid
proline
deoxyuridine
riboflavin
xanthosine
citrulline
7-methylguanosine
Glucose/fructose
1-methylhistidine
VIP scores
High
Low
Kidney
ccRCC
B
Histology
Site
Xanthine
Xanthosine
Deoxyuridine
Guanosine
Glycine
1-methyl-histidine
Nicotinamide
Myo-inositol
cADP-ribose
Taurine
Pyroglutamic acid
Homocysteine
Dimethylglycine
O-acetyl serine
5-Aminolevulinic acid
Creatine
GSH
S-lactoylglutathione
Spermidine
2-Hydroxyglutaric acid
1-Methyl nicotinamide
2-Isopropylmalic acid
Ascorbic acid
Valine
Acetylcholine
Carnitine
Propionylcholine
Carnitine-C6
Acetyl-carnitine
Citrulline
Ng-dimethyl arginine
Glucose/Fructose
Lactate
Proline
N-acetyl alanine
Kynurenine
Pyruvate
Alanine
Homoserine
Hydroxy proline
Deoxyinosine
Phenyl alanine
Methionine sulfoxide
Ornithine
Lysine
Tryptophan
2-Deoxycytidine
Cytidine
Alpha-ketoglutarate
UDP-glucuronic acid
Histology
CC
FH Deficient RCC
Normal
pRCC
tRCC
uRCC
Site
Kidney
Lymphnode
Met
Primary
D
class
Human ccRCC
Human Kidney
Mouse ccRCC
Mouse Kidney
Vitamin B2
Xanthosine
3-Methylhistidine
Uracil
Uridine
Hypoxanthine
Xanthine
Glycerate
N-6-trimethyllysine
Adenosine
Guanosine
Nicotinamide
Pipecolate
Urea
Gluconate
Glycine
Taurine
Aspartate
Serine
Glucuronate
GABA
Glycerophosphorylcholine
Dimethylglycine
Myoinositol
Homocysteine
NAD
Arginine
SAH
Cysteine
Putrescine
Homoserine
Succinate
Adenine
MTA
2-Aminoadipate
AMP
Glutamate
5'-CMP
Cystine
5-oxoproline
Cis-aconitate
Sedoheptulose-7-phosphate
Histidine
Asparagine
Methionine
Tyrosine
Phenylalanine
Valine
Isoleucine
Tryptophan
N6-acetyllysine
Dimethylarginine
Cytidine
Alanine
Lysine
Ornithine
Nicotinate
Trans-4-hydroxyproline
Acetylcarnitine
GSH
Spermidine
2-Hydroxyglutarate
2-Hydroxybutyrate
Creatine
Kynurenine
2'-deoxyinosine
Proline
Lactate
N1-methyladenosine
Creatinine
Pantothenate
Glutamine
Citrulline
Guanidinoacetate
Ascorbate
2-isopropylmalate

Figure S1. Metabolic alterations in RCC tumorgrafts and similarity between tumorgrafts and published human studies.

(A). Principal component analysis (PCA) plot of metabolomics data generated from 28 distinct RCC tumorgrafts passaged in NOD-SCID mice. Metabolomics data were logtransformed and median normalized to assess metabolic differences between normal kidney tissues and RCC subtypes using Metaboanalyst.

(B). Heatmap of the top 50 differential metabolites between RCC subtypes and kidney tissues, as assessed by one-way ANOVA. Metabolites of central carbon metabolism and glutathione metabolism are shown in red. The heatmap was generated using default parameters in Metaboanalyst.

(C). Variable importance in projection (VIP) plot of the top 20 metabolites differing between ccRCC and kidney using partial least squares-discriminant analysis (PLS-DA) in Metaboanalyst. Metabolites related to glutamine and glutathione metabolism are in red, and metabolites related to nucleotides are in blue.

(D). Heatmap of 76 common metabolites between tumorgrafts and published human ccRCC data (*17*). Raw data were log-transformed and median-normalized to combine and generate the heatmap using the default parameter in Metaboanalyst.

Figure S2

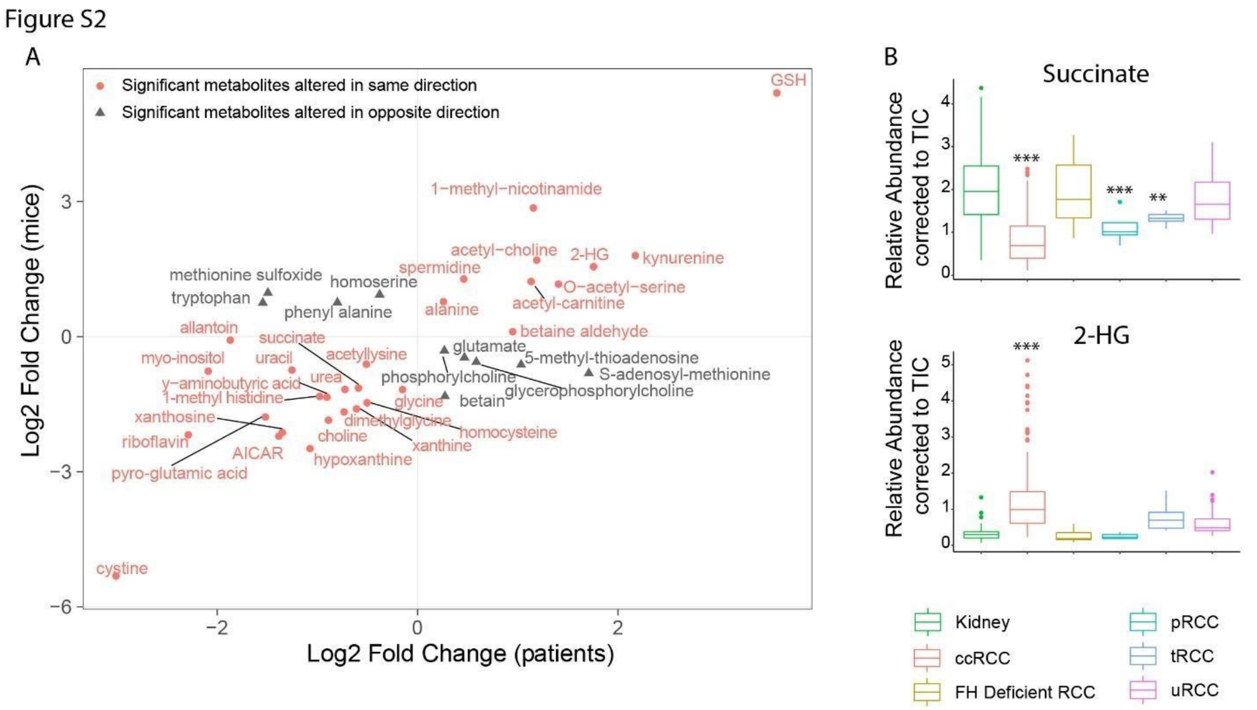

Figure S2. Correlation of metabolic features between ccRCC tumorgrafts and human studies and metabolic features of RCC tumorgrafts.

(A). Correlation plot of the metabolites altered in both ccRCC tumorgrafts and published human ccRCC data (*33*). In both studies, differential metabolites between tumor and kidney tissues were calculated using Student's t-test and corrected for FDR (Q value<0.05). Fold change was used as a surrogate for z-score to generate the correlation plot using ggplot in R. Positive Log2 fold change indicates metabolite elevation and negative Log2 fold change indicates metabolite depletion in tumors compared to kidney. Metabolites in red are altered in the same direction in tumorgrafts and patients, whereas metabolites in gray are altered in the opposite direction in tumorgrafts and patients.

(B). Boxplot of the relative abundance of succinate and 2-hydroxyglutarate in RCC tumorgrafts of different histological types, including ccRCC, FH deficient RCC, papillary RCC (pRCC), translocation RCC (tRCC), and unclassified RCC (uRCC). One-Way

ANOVA coupled with pairwise t-test in R software was used to calculate the significance of the data and P values were FDR corrected.

P values: ***<0.001, **<0.01, *<0.05

Figure S3

A

Tumorgraft	Mutations	Histology	Previous treatment (patients)
XP258	*VHL*	Clear cell with sarcomatoid	None
XP296	*VHL*	Clear cell with sarcomatoid	Everolimus
XP374	*VHL, SETD2, TP53*	Clear cell with sarcomatoid	None
XP955	*VHL*	Clear cell	None
XP490	*VHL, TP53*	Clear cell with sarcomatoid	None

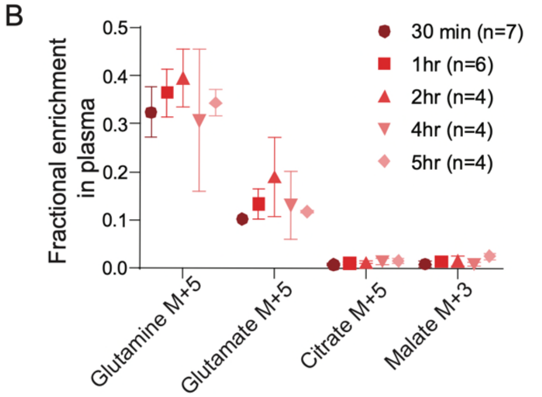

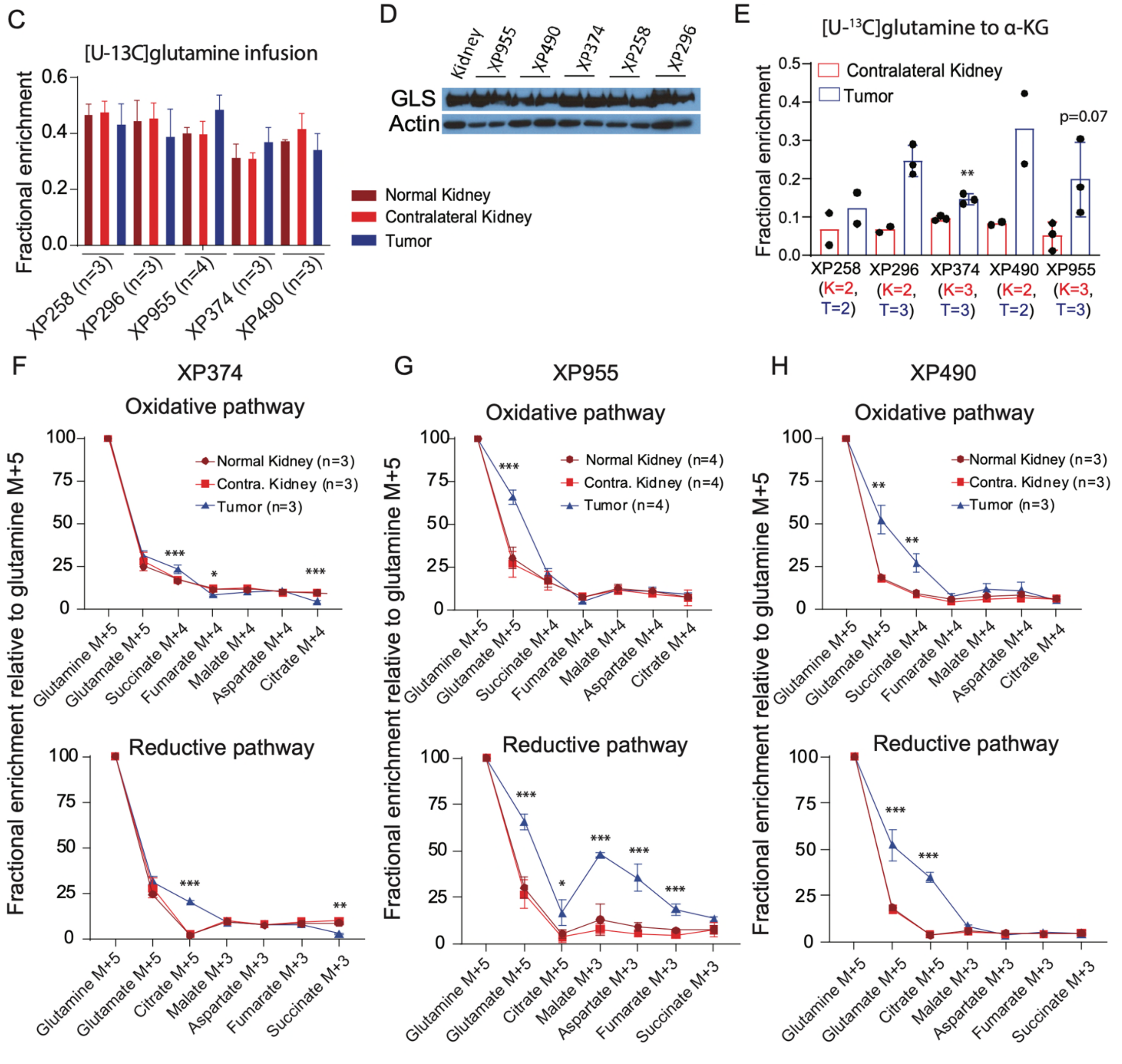

Figure S3. **Characterization of ^{13}C enrichment in ccRCC tumorgrafts infused with [U-^{13}C]glutamine.**

(A). Table showing genetic and histological characteristics of five ccRCC tumorgrafts used in this study. Four tumorgrafts were from treatment-naïve patients and one was from a patient previously treated with everolimus. Mutations in tumors from both patient and tumorgraft tissues were confirmed using whole-exome sequencing.

(B). Fractional enrichment of glutamine M+5, glutamate M+5, citrate M+5, and malate M+3 in plasma collected at different time points from mice infused with [U-^{13}C]glutamine.
(C). Fractional enrichment of ^{13}C-glutamine in the tumor, normal kidney and contralateral kidney collected from mice infused with [U-^{13}C]glutamine.

(D). Western blot showing glutaminase (GLS) expression relative to β-actin in contralateral kidney and tumor tissues collected from mice bearing orthotopic ccRCC tumorgrafts.

(E). Fractional enrichment of ^{13}C-labeled α-ketoglutarate (α-KG) M+5 in contralateral kidney and tumorgrafts collected from mice infused with [U-^{13}C]glutamine. The number of independent tissues is displayed as K=n for the kidney and T=n for the tumorgraft. Oneway ANOVA was used to determine statistical significance.

(F). Percentage enrichment of TCA cycle intermediates relative to [U-^{13}C]glutamine in XP374 tumorgraft, adjacent benign (normal kidney) and contralateral kidney. The top panel shows ^{13}C labeling via the oxidative pathway and the bottom panel shows ^{13}C labeling via the reductive pathway. The experiment was conducted in a minimum of 3

mice per tumorgraft model. One-way ANOVA was used to assess the statistical significance of ^{13}C enrichment between tissues.

(G). Same as in E, but for XP955 tumorgrafts.

(H)**.** Same as in E, but for XP490 tumorgrafts.

P values: ***<0.001, **<0.01, *<0.05

Figure S4

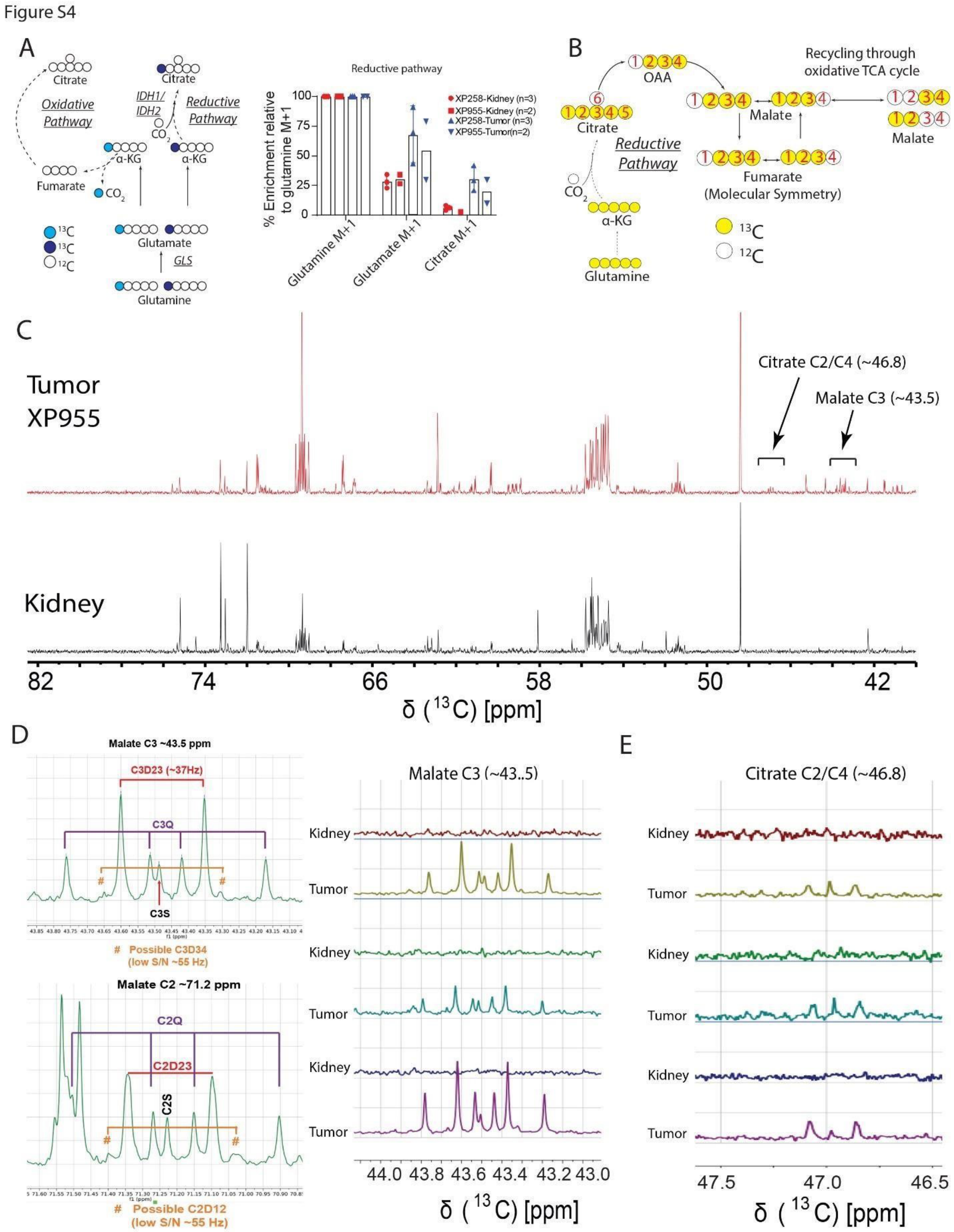

Figure S4
A
Citrate
Oxidative Pathway
IDH1/ IDH2
Reductive Pathway
α-KG
Fumarate
CO2
13C
13C
12C
Glutamate
GLS
Glutamine
Reductive pathway
% Enrichment relative to glutamine M+1
XP258-Kidney (n=3)
XP955-Kidney (n=2)
XP258-Tumor (n=3)
XP955-Tumor(n=2)
Glutamine M+1
Glutamate M+1
Citrate M+1
B
OAA
Citrate
Reductive Pathway
Recycling through oxidative TCA cycle
Malate
Fumarate (Molecular Symmetry)
13C
12C
Glutamine
C
Tumor XP955
Kidney
Citrate C2/C4 (~46.8)
Malate C3 (~43.5)
δ (13C) [ppm]
D
Malate C3 ~43.5 ppm
C3D23 (~37Hz)
C3Q
C3S
Possible C3D34 (low S/N ~55 Hz)
Malate C2 ~71.2 ppm
C2Q
C2D23
C2S
Possible C2D12 (low S/N ~55 Hz)
Malate C3 (~43..5)
Kidney
Tumor
δ (13C) [ppm]
E
Citrate C2/C4 (~46.8)
Kidney
Tumor
δ (13C) [ppm]

Figure S4. **GC-MS and NMR in ccRCC tumorgrafts infused with [U-^{13}C]glutamine.**

(A). Left panel shows a schematic of ^{13}C labeling from [1-^{13}C]glutamine. The light blue-filled circles show ^{13}C labeling via the oxidative pathway, and the dark blue-filled circles show ^{13}C labeling via the reductive pathway. Citrate is labeled via the reductive pathway. The right panel shows the percentage enrichment of ^{13}C in glutamine M+1, glutamate M+1, and citrate M+1 relative to [1-^{13}C]glutamine in tumor and contralateral kidney from two tumorgraft models (XP258, n=3; XP955, n=2).

(B). Schematic of ^{13}C positions in TCA cycle intermediates via the reductive pathway. The yellow circles represent ^{13}C, and the numbers in the circles represent the position. Although reductive carboxylation initially generates malate and fumarate labeled with ^{13}C at positions 2, 3 and 4 (234), 123 labeling equilibrates with 234 labeling due to molecular symmetry of fumarate. Labeling at 12 and 34 in malate arise from processing along the oxidative pathway.

(C). ^{13}C NMR spectra of an XP955 tumorgraft (red) and contralateral kidney (black) collected from mice infused with [U-^{13}C]glutamine. The spectra corresponding to citrate C2/C4 (~46.8 ppm) and malate C3 (~43.5 ppm) are highlighted. These spectra are representative of 3 biological experiments.

(D). In the left panel, malate C3 and C2 spectra are shown, highlighting peaks for singlets (S: C3S or C2S), doublets (D: C3D23 and C3D34; or C2D12 and C2D23), and quartets (Q: C3Q or C2Q). The predicted ppm for C3D34 and C2D12 multiplets generated via the oxidative TCA cycle are highlighted in orange. The right panel shows spectra for malate C3 (~43.5 ppm) across all samples (n=3 tumors and 3 kidneys).

(E). Spectra for citrate C2/C4 (~46.8 ppm) for all samples (n=3 tumors and 3 kidneys).

Figure S5

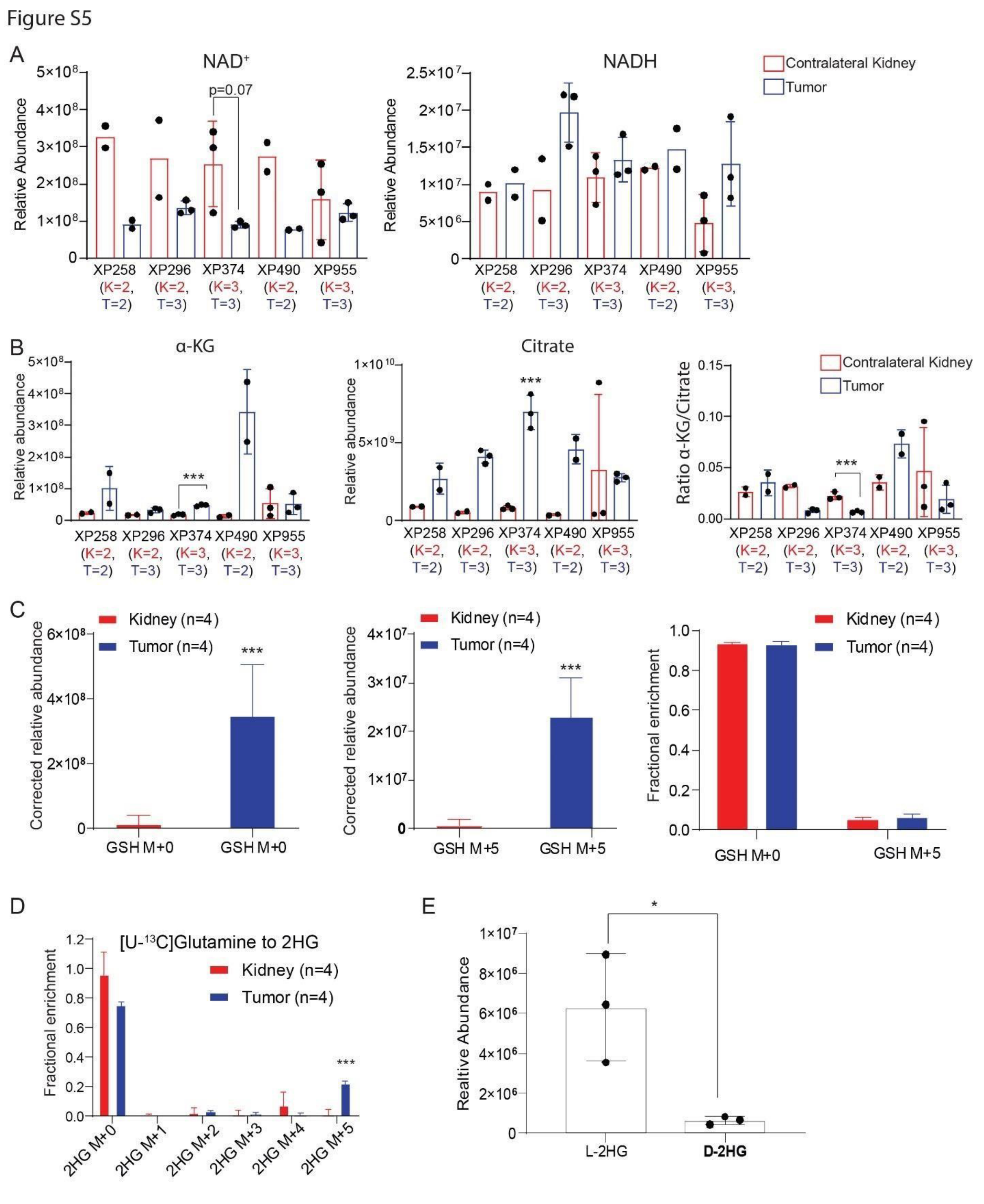
Figure S5
A
NAD+
p=0.07
NADH
Contralateral Kidney
Tumor
Relative Abundance
XP258 (K=2, T=2)
XP296 (K=2, T=3)
XP374 (K=3, T=3)
XP490 (K=2, T=2)
XP955 (K=3, T=3)
B
α-KG
Citrate
Relative abundance
Ratio α-KG/Citrate
C
Kidney (n=4)
Tumor (n=4)
Corrected relative abundance
Fractional enrichment
GSH M+0
GSH M+5
D
[U-13C]Glutamine to 2HG
Kidney (n=4)
Tumor (n=4)
Fractional enrichment
2HG M+0
2HG M+1
2HG M+2
2HG M+3
2HG M+4
2HG M+5
E
Realtive Abundance
L-2HG
D-2HG

Figure S5. Assessment of the relative abundance of NAD^+, NADH, citrate, and αKG, and the fractional enrichment of GSH, and 2HG levels in ccRCC tumorgrafts infused with [U-^{13}C]glutamine.

(A). Relative abundance of NAD^+ and NADH in ccRCC tumorgrafts and contralateral kidney, normalized to protein abundance. The number of tissues used for analysis is shown below each plot as K=n for kidney and T=n for tumor. One-way ANOVA was used to determine statistical significance.

(B). Relative abundance of α-KG, citrate, and α-KG/citrate ratio in ccRCC tumorgrafts and contralateral kidneys. The number of tissues used for the analysis is shown below each plot as K=n for kidney and T=n for tumor. One-way ANOVA was used to determine statistical significance.

(C). Corrected abundance and fractional enrichment of M+0 and M+5 ^{13}C-labeled glutathione (GSH) in tumors (XP955) and kidneys of mice infused with [U-^{13}C]glutamine. Student's t-test was used to determine the p-value.

(D). Mass isotopologues of 2HG in tumor (XP955) and kidney of mice infused with [U^{13}C]glutamine. One-way ANOVA was used to determine the p-value.

(E). Relative abundance of L-2HG and D-2HG isoforms in ccRCC tumorgrafts (XP296). Student's t-test was used to calculate the p-value.

P values: ***<0.001, **<0.01, *<0.05

Figure S6

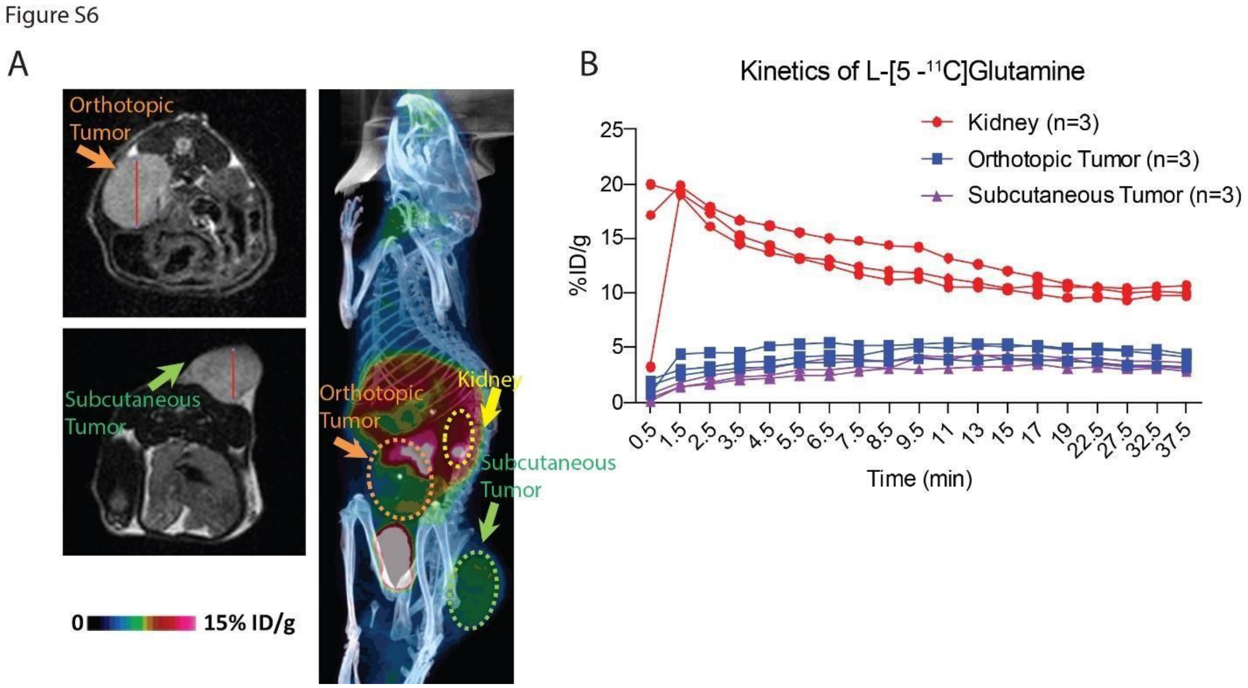

Figure S6. **MRI and L-[5-^{11}C]glutamine PET/CT analysis in ccRCC tumorgrafts.**

(A). MRI and PET/CT of mice bearing both orthotopic and subcutaneous XP490 tumorgrafts (n=3 mice). MRI was used to localize the relevant tissues in these imaging planes. PET/CT images show the signal from L-[5-^{11}C]glutamine for kidney and tumors.

(B). Kinetics of L-[5-^{11}C]glutamine displayed as % injected dose per gram of mouse (%ID/g) in the kidney (red), orthotopic tumor (blue), and subcutaneous tumor (purple). Data plotted from n=3 mice.

Figure S7

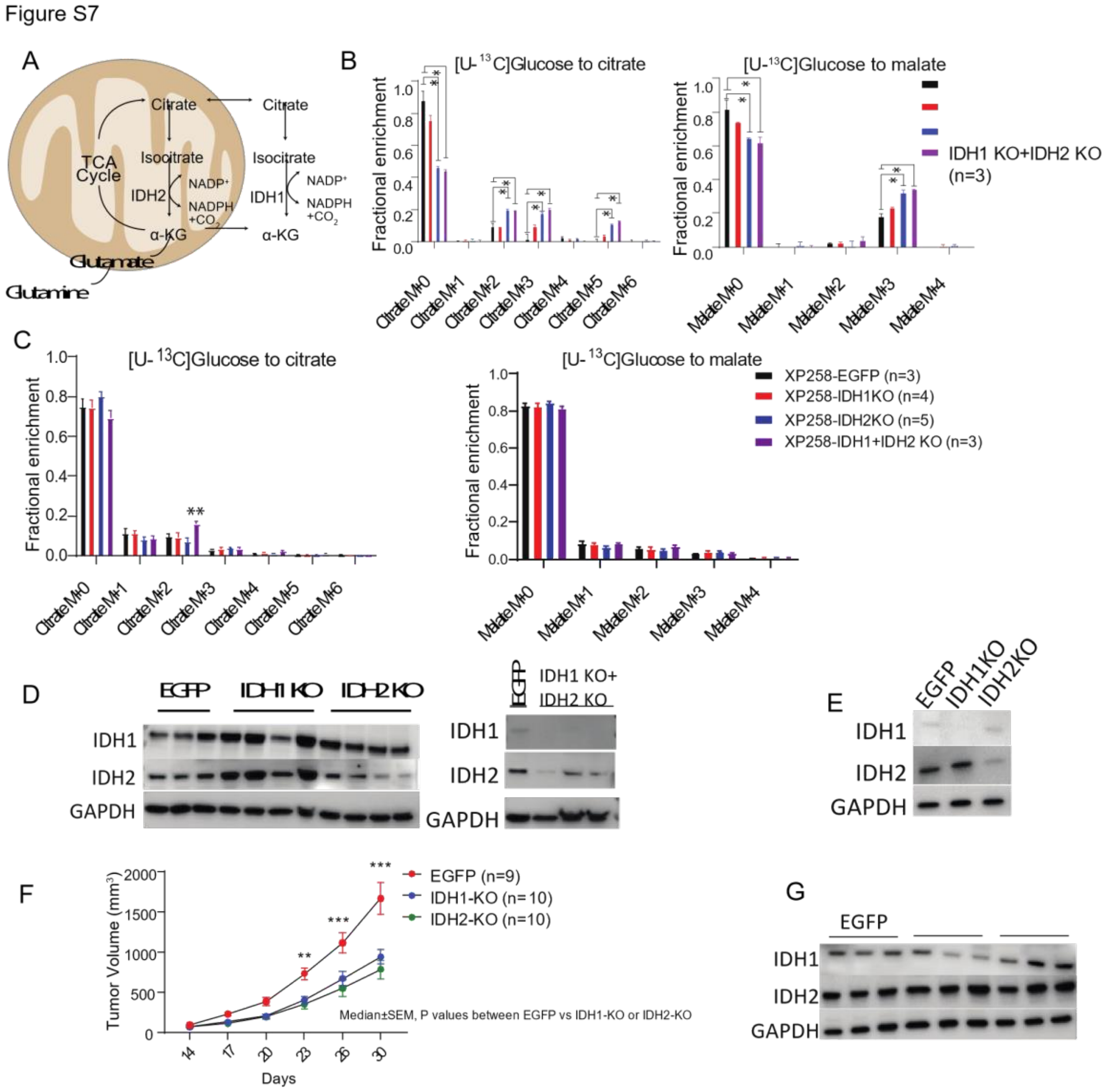
Figure S7
A
Citrate
Citrate
TCA Cycle
Isocitrate
Isocitrate
IDH2
NADP+
NADPH +CO2
IDH1
NADP+
NADPH +CO2
α-KG
α-KG
Glutamate
Glutamine
B
[U-13C]Glucose to citrate
Fractional enrichment
Citrate M+0
Citrate M+1
Citrate M+2
Citrate M+3
Citrate M+4
Citrate M+5
Citrate M+6
[U-13C]Glucose to malate
Malate M+0
Malate M+1
Malate M+2
Malate M+3
Malate M+4
IDH1 KO+IDH2 KO (n=3)
C
[U-13C]Glucose to citrate
[U-13C]Glucose to malate
XP258-EGFP (n=3)
XP258-IDH1KO (n=4)
XP258-IDH2KO (n=5)
XP258-IDH1+IDH2 KO (n=3)
D
EGFP
IDH1 KO
IDH2 KO
IDH1
IDH2
GAPDH
EGFP
IDH1 KO+ IDH2 KO
E
EGFP
IDH1KO
IDH2KO
F
Tumor Volume (mm3)
Days
EGFP (n=9)
IDH1-KO (n=10)
IDH2-KO (n=10)
Median±SEM, P values between EGFP vs IDH1-KO or IDH2-KO
G
EGFP
IDH1
IDH2
GAPDH

Figure S7. IDH1 and IDH2 KO rewire substrate oxidation in TCA cycle and reduce tumor growth in ccRCC.

(A). Schematic of $NADP^+$/NADPH-dependent IDH1 (cytosol) and IDH2 (mitochondria) reactions that interconvert isocitrate and α-KG. Glutamine-derived α-KG is reductively carboxylated by NADPH-dependent IDH1 and IDH2 in cytosol and mitochondria, respectively.

(B). Fractional enrichment of ^{13}C-labeled isotopologues of citrate and malate in XP258derived EGFP, IDH1 KO, IDH2 KO, and IDH1 KO+IDH2 KO cells labeled with [U^{13}C]glucose for 3 hours. One-way ANOVA was used to assess the statistical significance of ^{13}C enrichment in isotopologues of citrate and malate among the cell lines (n=3).

(C). Fractional enrichment of ^{13}C-labeled isotopologues of citrate and malate in xenografts derived from EGFP, IDH1 KO, IDH2 KO, and IDH1 KO+IDH2 KO cells. The mice were infused with [U-^{13}C]glcuose for 3 hours before harvesting the tissues for GC/MS analysis. One-way ANOVA was used to assess the statistical significance of ^{13}C enrichment in isotopologues of citrate and malate (n=3).

(D). Protein expression of IDH1 and IDH2 in tissues collected at the end of the tumor growth study. GAPDH was used as a loading control.

(E). Western blot showing the expression of IDH1 and IDH2 protein in CRISPRCas9generated pools containing either EGFP-targeting gRNA or gRNA targeting IDH1 or IDH2. The parental cells were an independent XP258-derived cell line from the one shown in the main figure, derived by a different lab.

(F). Growth of tumors derived from control cells and IDH1 and IDH2 KO cells (n=5 female and 5 male mice in each group, except for 5 male and 4 female mice in EGFP) shown in panel E. One-way ANOVA was used to assess the statistical significance at each time point of tumor measurement.

(G). Western blot showing the expression of IDH1 and IDH2 in tumors from panel F.

P values: ***<0.001, **<0.01, *<0.05

Figure S8

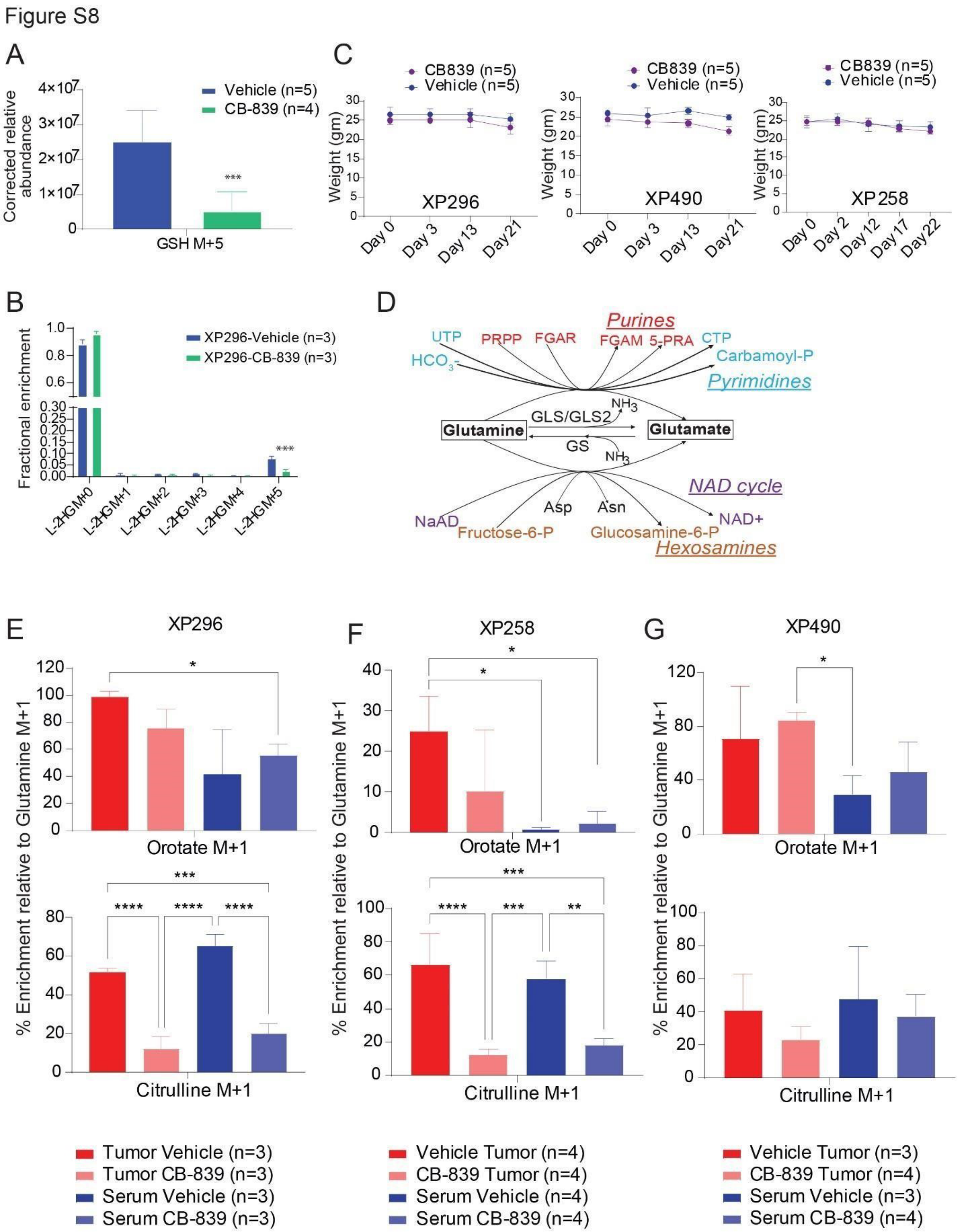

Figure S8
A
Corrected relative abundance
Vehicle (n=5)
CB-839 (n=4)
GSH M+5
C
CB839 (n=5)
Vehicle (n=5)
Weight (gm)
XP296
XP490
XP258
Day 0
Day 3
Day 13
Day 21
Day 2
Day 12
Day 17
Day 22
B
Fractional enrichment
XP296-Vehicle (n=3)
XP296-CB-839 (n=3)
D
Purines
UTP
PRPP
FGAR
FGAM
5-PRA
CTP
HCO3-
Carbamoyl-P
Pyrimidines
GLS/GLS2
NH3
Glutamine
Glutamate
GS
NAD cycle
Asp
Asn
NaAD
NAD+
Fructose-6-P
Glucosamine-6-P
Hexosamines
E
XP296
F
XP258
G
XP490
% Enrichment relative to Glutamine M+1
Orotate M+1
Citrulline M+1
Tumor Vehicle (n=3)
Tumor CB-839 (n=3)
Serum Vehicle (n=3)
Serum CB-839 (n=3)
Vehicle Tumor (n=4)
CB-839 Tumor (n=4)
Serum Vehicle (n=4)
Serum CB-839 (n=4)
Vehicle Tumor (n=3)
CB-839 Tumor (n=4)
Serum Vehicle (n=3)
Serum CB-839 (n=4)

Figure S8. Effects of CB-839 on glutamine-dependent metabolism in ccRCC in vivo.

(A). Effect of CB-839 on relative abundance of labeled GSH in XP296 tumorgrafts.

(B). Effect of CB-839 on ^{13}C labeling in L-2HG (L-2 hydroxyglutarate) in XP296 tumorgrafts. Student's t-test was used to assess the p-value for data represented in panels A and B.

(C). Weight of mice treated with vehicle or CB-839 (200mg/kg twice daily) during tumor growth study.

(D). Conversion of glutamine to glutamate involves glutaminases (GLS and GLS2) and amidotransferases.

(E). Enrichment relative to glutamine M+1 ([amide-^{15}N]glutamine) for orotate and citrulline in plasma and XP296 tumors collected from vehicle or CB-839 treated mice. Mice bearing subcutaneous tumors were treated with 7 doses of either vehicle or CB-839 (200mg/kg twice daily) before infusion with [amide-^{15}N]glutamine. Fractional enrichment of each metabolite was corrected to the fractional enrichment of [amide-^{15}N]glutamine. P values were calculated using one-way ANOVA.

(F). Same as in E, but for the XP258 tumorgrafts.

(G). Same as in E, but for XP490 tumorgrafts.

P values: ****<0.0001, ***<0.001, **<0.01, *<0.05

Figure S9

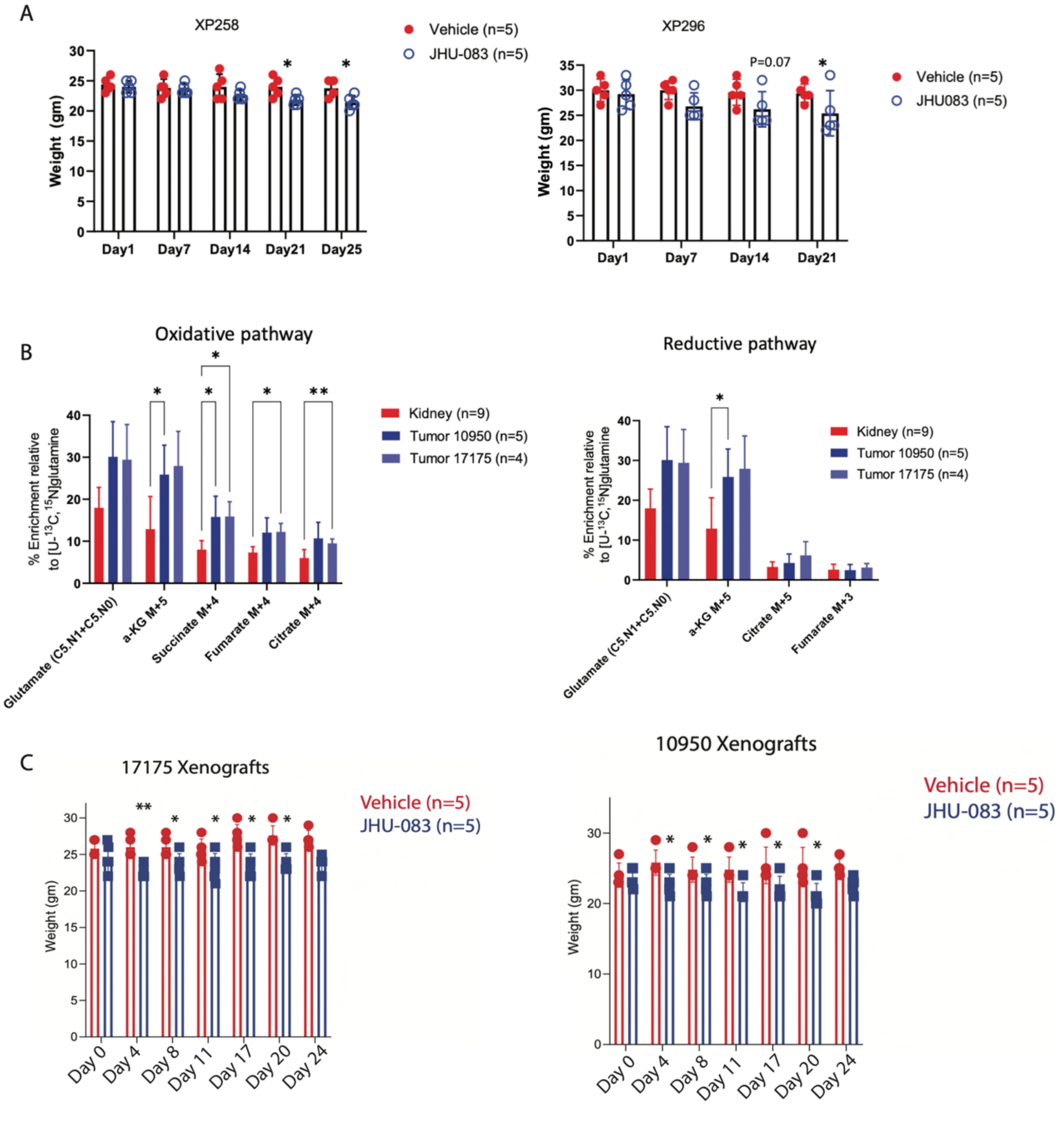
A
XP258
Vehicle (n=5)
JHU-083 (n=5)
Weight (gm)
Day1
Day7
Day14
Day21
Day25
XP296
P=0.07
Vehicle (n=5)
JHU083 (n=5)
B
Oxidative pathway
% Enrichment relative to [U-13C, 15N]glutamine
Glutamate (C5.N1+C5.N0)
a-KG M+5
Succinate M+4
Fumarate M+4
Citrate M+4
Kidney (n=9)
Tumor 10950 (n=5)
Tumor 17175 (n=4)
Reductive pathway
Citrate M+5
Fumarate M+3
C
17175 Xenografts
Vehicle (n=5)
JHU-083 (n=5)
Day 0
Day 4
Day 8
Day 11
Day 17
Day 20
Day 24
10950 Xenografts

Figure S9: Effect of JHU-083 on mouse weight and metabolite labeling from [U^{13}C]glutamine in RCC allografts derived from syngeneic mouse cell lines.

(A). Effect of JHU-083 treatment on the weight of NOD-SCID mice bearing XP258 and XP296 tumorgrafts. Mice were treated i.p. with 1.83 mg/kg of JHU-083.

(B). Percent enrichment of TCA cycle intermediates relative to [U-^{13}C,^{15}N]glutamine in tumors derived from 10175 and 10950 cells in immunocompetent mice. ANOVA was used to determine the p-value.

(C). Weight of mice bearing 17175 and 10950 xenografts treated with JHU-083 (0.915mg/kg, via i.p injection). ANOVA was used to determine the significance of weight loss in the animals.

.

Table S1: Clinical characteristics of 28 RCC tumorgrafts.

**Characteristic**	**N = 28**[1]
Previous.Therapies	
Everolimus	1 (3.6%)
HD-IL2, Interferon, Bevacizumab	1 (3.6%)
Sunitinib	3 (11%)
Treatment Naïve	23 (82%)
Histological.Subtype	
ccRCC	19 (68%)
FH Deficient RCC	2 (7.1%)
pRCC	2 (7.1%)
tRCC	1 (3.6%)
uRCC	4 (14%)
Grade	
3	11 (44%)
4	13 (52%)
High grade	1 (4.0%)
Unknown	3
Source	
Metastasis	5 (18%)
Primary Tumor	22 (79%)
Regional Lymphnode	1 (3.6%)
VHL	
Frameshift Indel	6 (25%)
Missense	5 (21%)
Nonsense	1 (4.2%)
Nonstop	1 (4.2%)
WT	11 (46%)
Unknown	4

[1] n (%)

Supplementary Data S1: Total Ion count normalized metabolomics data from RCC tumorgrafts

Supplementary Data S2: Clinical characteristics of 28 RCC tumorgrafts used for metabolomics study
